# Does Speciation between *Arabidopsis halleri* and *Arabidopsis lyrata* Coincide with Major Changes in a Molecular Target of Adaptation?

**DOI:** 10.1371/journal.pone.0026872

**Published:** 2011-11-01

**Authors:** Camille Roux, Vincent Castric, Maxime Pauwels, Stephen I. Wright, Pierre Saumitou-Laprade, Xavier Vekemans

**Affiliations:** 1 Université Lille Nord de France, Lille, France; 2 FRE 3268 CNRS Université Lille 1, Villeneuve d'Ascq, France; 3 Department of Ecology and Evolutionary Biology, University of Toronto, Toronto, Canada; University of Umeå, Sweden

## Abstract

Ever since Darwin proposed natural selection as the driving force for the origin of species, the role of adaptive processes in speciation has remained controversial. In particular, a largely unsolved issue is whether key divergent ecological adaptations are associated with speciation events or evolve secondarily within sister species after the split. The plant *Arabidopsis halleri* is one of the few species able to colonize soils highly enriched in zinc and cadmium. Recent advances in the molecular genetics of adaptation show that the physiology of this derived ecological trait involves copy number expansions of the *AhHMA4* gene, for which orthologs are found in single copy in the closely related *A. lyrata* and the outgroup *A. thaliana*. To gain insight into the speciation process, we ask whether adaptive molecular changes at this candidate gene were contemporary with important stages of the speciation process. We first inferred the scenario and timescale of speciation by comparing patterns of variation across the genomic backgrounds of *A. halleri* and *A. lyrata*. Then, we estimated the timing of the first duplication of *AhHMA4* in *A. halleri*. Our analysis suggests that the historical split between the two species closely coincides with major changes in this molecular target of adaptation in the *A. halleri* lineage. These results clearly indicate that these changes evolved in *A. halleri* well before industrial activities fostered the spread of Zn- and Cd-polluted areas, and suggest that adaptive processes related to heavy-metal homeostasis played a major role in the speciation process.

## Introduction

Ever since Darwin [1] introduced the idea that natural selection may be the driving force behind the origin of species, the role of adaptive processes at play during speciation has remained controversial. One approach has tried to catch speciation *in flagrante delicto* by focusing on partially reproductively isolated ecotypes, asking how ecology and genetics interact and cause the evolution of reproductive barriers [2], [3]. While this approach is well suited for investigating the modes of speciation, and in particular for revealing the ecological speciation process, its validity has been questioned because there is no guarantee that the studied ecotypes will ever attain species status. Hence, a different, “retrospective” approach studies well-established species among which reproductive isolation is complete. These studies are able to determine the genetics of extant reproductive barriers, but the modes of speciation, and in particular the role of divergent selection in the early phases of the speciation process, are notoriously difficult to infer *a posteriori* [3].

Recent developments in population genomic tools have brought new prospects for the retrospective approach, making it possible to study the divergence process *a posteriori* by estimating parameters under simple demographic models of speciation [4], [5]. In particular, the recently developed approximate Bayesian computation (ABC) approach provides a framework for testing alternative demographic models of speciation [6], [7], and also allows great flexibility in the type of models that can be compared [8]. In parallel, the availability of genomic tools in model species along with population genomic and candidate gene approaches have resulted in the identification of major genes and molecular processes that drive ecological specialization within or between species [9]. Such knowledge may ultimately help understand the chronology of evolutionary genetic processes underlying the response of species and organisms to their natural environment. Strikingly, these two lines of advances have rarely been integrated, and the demographical and historical contexts of most documented ecological adaptations remain poorly characterized. In particular, it remains largely unknown whether key divergent ecological adaptations are indeed associated with speciation events or evolve secondarily within sister species after the split.

Here, we investigated the ecological speciation process using a retrospective approach by combining demographic inference on the timing of speciation with studies on a molecular target of adaptation. We focused on the pair of plant species *Arabidopsis halleri* and *A. lyrata* (Brassicaceae), two close relatives of the model species *A. thaliana* from which they diverged about 5 MY [10], or earlier [11]. *A. halleri* is mainly distributed in continental Europe, although a subspecies (*A. halleri* ssp. *gemmifera*) with a disjunct distribution occurs in Eastern Eurasia [10]. In comparison, *A. lyrata* has a circumboreal distribution but also occurs in Western and Central Europe [10]. The two species differ in an important ecological trait. *A. halleri* is a pseudometallophyte species able to colonize soils highly enriched in zinc and cadmium, and can accumulate these metals in its aerial parts [12], [13]. *A. lyrata* and the outgroup *A. thaliana* are both non-accumulators and sensitive to zinc and cadmium, strongly suggesting that zinc and cadmium tolerance and hyperaccumulation in *A. halleri* are derived ecological traits. Moreover, all data available today indicate that these traits are shared by populations growing on metalliferous as well as non-metalliferous soils, species-wide [12], [14]. This observation raises the question of the role of human (industrial) activities on selection of such phenotypes. According to one scenario, recent heavy metal pollution due to industrial activities could have been the main selection pressure leading to changes in metal homeostasis in the *A. halleri* lineage. Hence, populations presently growing on non-metalliferous soils would have evolved recently from metallicolous populations, suggesting the occurrence of a recent genetic bottleneck in *A. halleri*. An alternative scenario would be the early fixation in the *A. halleri* lineage of mutations inducing changes in metal homeostasis well before the pollution induced by human activities.

Recently, one gene has been characterized as a key factor involved in zinc homeostasis in *A. halleri*. *HMA4* (*heavy metal ATPase 4*) encodes a metal pump controlling root-to-shoot Zn transport by loading Zn into xylem vessels [15]. This gene has a strikingly high transcript level in *A. halleri*, as the result of *cis*-regulatory changes and tandem triplication. RNA silencing of *HMA4* in *A. halleri* provides strong support that these changes play a major role in Zn and Cd tolerance and hyperaccumulation in this species [15]. Moreover, independent tandem duplications of *HMA4* also occurred in *Noccaea caerulescens*, another Zn and Cd hyperaccumulator species [16], reinforcing the role of duplication-mediated increased expression of this gene in the evolution towards metal tolerance and hyperaccumulation.

In this paper, we tested whether the adaptive molecular changes at this gene are contemporary with important stages of the speciation process. We first compared patterns of genetic variation across the genomic backgrounds of *A. halleri* and *A. lyrata* to test alternative demographic models of speciation. Then, we estimated the timing of the first duplication of *AhHMA4* in the *A. halleri* lineage. Our analysis supports that the evolution of Zn and Cd tolerance in *A. halleri* was not followed by a strong bottleneck. Moreover, the historical split between *A. halleri* and *A. lyrata* closely coincides with the evolution of major changes in metal homeostasis in the *A. halleri* lineage. These results clearly indicate that these changes evolved in *A. halleri* well before the spread of Zn-and Cd-polluted areas through industrial activities, and suggest that adaptive processes related to heavy-metal homeostasis have occurred during the speciation process.

## Results

**Figure 1:**
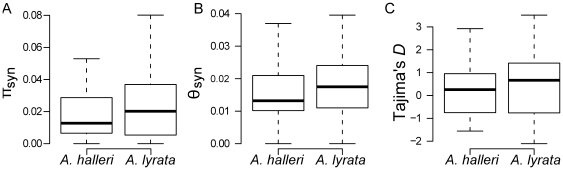
Box-plots of diversity and Tajima's *D* statistics for *A. halleri* and *A. lyrata*. (*A*) synonymous nucleotide diversity, π_syn_; (*B*) Watterson's θ_syn_ statistic; (*C*) Tajima's *D* statistic.

To evaluate the demographic and historical context of speciation, we estimated the levels of nucleotide diversity in the genomic background of *A. halleri* and *A. lyrata*. In *A. halleri*, we resequenced 29 unlinked nuclear genes (totaling 26 kb of coding sequence per individual, Table S1) on a geographically broad sample of 31 individuals from five European populations. In *A. lyrata*, we used published sequence data [17] for the orthologs in 48 individuals from four European populations. Over both species, we observed a total of 850 biallelic polymorphic sites (Table S2). Levels of synonymous polymorphism estimated at these loci were very similar in both species based on either the nucleotide diversity statistic, π [18] (π*_syn_* = 0.0206 *vs.* 0.0240, for *A. halleri* and *A. lyrata* respectively; Fig. 1*A*, Table S3) or Watterson's *θ_W_* statistic [19] (*θ_W-syn_* = 0.0174 *vs.* 0.0190; Fig. 1*B*, Table S3), and the differences were not significant (Wilcoxon signed-rank test, *W* = 383, *P* = 0.5650 for π*_syn_*; and *W* = 368, *P* = 0.4187 for *θ_W-syn_*). Levels of synonymous *θ_W_* per nucleotide and per locus varied slightly among *A. halleri* populations, and ranged from 0.0108 (SD = 0.015) for the CZ population, to 0.0161 (SD = 0.016) for the Slovenian population (Table S4). The Tajima's estimator π*_syn_* measured per nucleotide and per locus ranged from 0.0114 (SD = 0.0187) to 0.0176 (SD = 0.0197). The site frequency spectrum, measured by Tajima's *D* [20] shows levels across loci around the neutral expectation of 0 (mean *D_hal_* = 0.239, mean *D_lyr_* = 0.513, *W* = 334, *P* = 0.3489) (Fig. 1*C*), suggesting no particular recent changes in population sizes.

**Figure 2:**
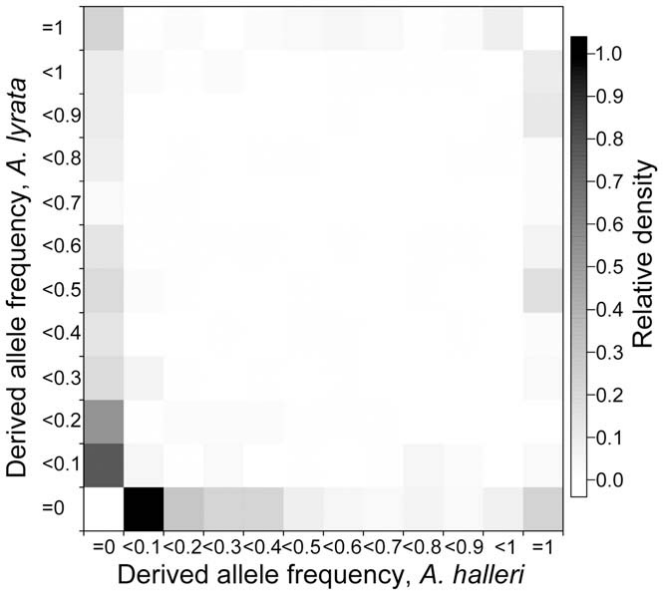
Distributions of derived synonymous SNP frequencies in *A. halleri* and A. *lyrata* calculated using *A. thaliana* as an outgroup. Exclusive polymorphic sites (bottom row and first column) are defined as positions where the derived allele frequency is between >0 and <1 in one species, but has a frequency of zero in the other species. Fixed differences are positions where the derived allele frequency is  = 0 in one species and  = 1 in the other species. Shared polymorphic sites are positions where the derived frequencies are >0 and <1 in both species. Putatively ancestral polymorphic sites are positions where the derived allele frequency is  = 1 in one species and between zero and unity in the other species.

The joint frequency spectra of derived synonymous sites in *A. halleri* and *A. lyrata* (Fig. 2), in reference to the outgroup *A. thaliana*, clearly did not support strong differentiation between the two species since only 7.2% of polymorphic sites were fixed for a derived allele in either species (Fig. S1). The total amount of putative ancestral polymorphisms contributed greatly to the observed level of diversity: 12.8% of all polymorphic sites were shared between the two species and 14.4% of sites showed polymorphisms in one species for a derived allele that was fixed in the other species (Sx*_hal_*f*_lyr_* = 5% and Sx*_lyr_*f*_hal_* = 9.4%, using the notation of [21]), giving a total of 27.2% of segregating polymorphisms being putatively of ancestral origin. Finally, a large amount of the observed polymorphism was private to each species (31.4% and 34.2% of all polymorphic sites in *A. halleri* and *A. lyrata* respectively, Fig. S1). Between-species differentiation measured by F_ST_ (Average = 0.4566, SD = 0.211) ranged across loci from 0.0688 for *At1g59720* to 0.8299 for *At1g06520* (Table S8).

**Figure 3:**
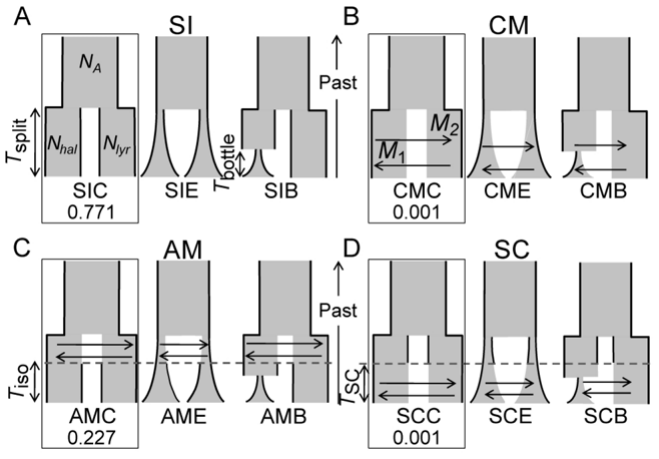
Alternative scenarios of speciation for *A. halleri* and *A. lyrata*. Four classes of scenarios according to the pattern of migration: strict isolation (SI), constant migration (CM), ancient migration (AM) and secondary contact (SC). Three alternative models within each class of scenarios: constant population size (SIC, CMC, AMC, SCC), exponential population growth (SIE, CME, AME, SCE) and bottleneck specific to *A. halleri* followed by exponential population growth (SIB, CMB, AMB, SCB). The migration rate *M* is expressed in 4 *Nm* units, where *m* is the proportion of a population made up of migrants from the other population per generation. *N* is the effective population size expressed in numbers of individuals. *A. halleri* (*N_hal_*), *A. lyrata* (*N_lyr_*), or the ancestor (*N_A_*). The posterior probabilities of the best model selected under each scenario are reported.

These high levels of putative ancestral polymorphisms in both species can be due to either incomplete lineage sorting or gene flow between species, although the almost complete absence of haplotype sharing among species provides support for the former hypothesis (data not shown). Using model choice procedures under an ABC framework, we could clearly reject scenarios with ongoing migration (Fig. 3, see also Text S1 for an account of the tests on the robustness of this result). Both models allowing for ongoing migration (the “constant migration” and the “secondary contact” models) had very low posterior probabilities (*P*<0.001, Fig. 3, Table S5). In contrast, the “strict isolation” and “ancient migration” models in which migration was assumed to have completely ceased, had high posterior probabilities, the former being better supported (*P* = 0.771 and 0.227, respectively). Using numerical simulations, we tested the robustness of the model choice procedure and found that a posterior probability of 0.771 for the strict isolation model was highly significant (*P* = 0.975; Text S1, Fig. S2). ABC analyses also clearly favored all models with no temporal variation in effective population size (Fig. 3, Table 1). We thus rejected the hypothesis that changes in metal homeostasis occurred only recently during colonization of polluted sites under strong selection for Zn and Cd tolerance, followed by colonization of non-metalliferous sites, processes that should have caused a recent genetic bottleneck in *A. halleri*. The lack of evidence for a recent genetic bottleneck in *A. halleri* was also suggested by multilocus analyses of nucleotide polymorphism in a single German population [22].

**Table 1:** For each class of scenarios (see Fig. 3), posterior probabilities of models with constant population size versus alternative models with exponential population growth or recent bottlenecks in *A. halleri*.

scenario	Posterior probabilities of constant population size models against:	
	exponential population growth models	recent botleneck in *A. halleri* models
SI	0.623 (0.794)	0.732 (0.950)
CM	0.699 (0.972)	0.831 (0.989)
AM	0.714 (0.951)	0.833 (0.974)
SC	0.652 (0.944)	0.736 (0.584)

Values in brackets represent the probabilities for each class of scenarios that the constant population size model (SIC, CMC, AMC, and SCC, see Fig. 3) is the correct model, given the observed posterior probabilities (see Text S1).

**Figure 4:**
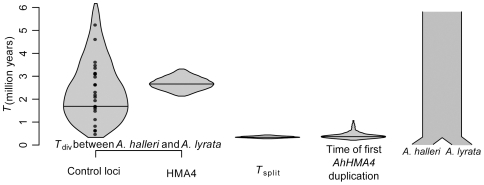
Coincidence of speciation time of *Arabidopsis halleri* and *A. lyrata* and the first duplication of the *AhHMA4* gene. Distributions show the 95% HPD of (1) the average time of divergence (*T_div_*) per locus between any *A. halleri* and *A. lyrata* lineages estimated as *T_div_* = *K*_syn_/(2.μ) where *K*_syn_ is the observed synonymous divergence per locus and μ the synonymous mutation rate- black dots represent the observed values at 29 loci; (2) the divergence time between *A. halleri* and *A. lyrata* gene copies at *HMA4* locus estimated with BEAST; (3) the time of speciation (*T_split_*) under the best model obtained using our ABC approach; and (4) the time of the first duplication at *AhHMA4* estimated with BEAST. Thick horizontal lines represent the mode of each distribution.

Parameter estimation under the best supported model (strict isolation with constant population size –SIC model) pointed to a more recent divergence ≈337,000 [272,800–438,200] years ago (Table 2, Fig. 4, Fig. S3) than the previous estimate of 2 MY old divergence [23], [24]. This discrepancy is due to a large difference between the time of species separation and the mean divergence time of *A. halleri* and *A. lyrata* gene copies at the 29 loci (Fig. 4), which is itself due to a large ancestral population size (≈533,000 individuals) as compared to that estimated for the current *A. halleri* (≈82,000) and *A. lyrata* (≈79,200) populations (Table 2).

**Table 2:** Demographic parameters estimated using ABC under the SIC (strict isolation model with constant population size).

*N_hal_* *	*N_lyr_* *	*N_A_* *	*T_split_* †
82	79.2	532.9	337.4
(65.2–98.9)	(65.2–103.9)	(440.2–657.7)	(272.8–438.2)

*Effective population size (expressed as 10^3^ individuals) for *A. halleri* (hal), *A. lyrata* (lyr), and their ancestor (A).

† Time (ka) of the split between *A. halleri* and *A. lyrata*.

The 95% highest posterior density intervals are shown in parentheses.

We then compared the inferred speciation times with the timing of copy number expansion of *AhHMA4*, contributing to drastic changes in metal homeostasis in *A. halleri* [15], [25]. To obtain time estimates for this event, we compared paralogous nucleotide sequences in *A. halleri* with orthologous sequences in *A. thaliana* and *A. lyrata*. Because gene conversion can bias molecular clocks, we first ensured that it did not occur at *AhHMA4* [26]. Then, we checked that all paralogous sequences of *AhHMA4* in *A. halleri* clustered together, *i.e.* that the single copy gene in *A. lyrata* appeared as an outgroup sequence (Fig. S4). Finally, we estimated the time of the first duplication event. Our estimate indicated that it occurred ≈357,000 [216,968–1,057,370] years ago, suggesting that it was contemporary with the speciation between *A. halleri* and *A. lyrata* (Fig. 4). The second *AhHMA4* duplication was estimated to have occurred ≈100,000 years after the first duplication event, e.g. ≈250,000 [5,790–474,510] years ago.

## Discussion

Research on the genetics of speciation has mainly focused on the detection of secondary Dobzhansky-Muller genetic incompatibilities that reduce the probability of gene exchange between extant species (e.g. [27], [28], [29], [30]). Although equally important, the initial causes of divergence remain much more poorly documented at the genetic and molecular level [31]. By combining molecular genetics of adaptation approaches with population genomic approaches, we found that a major adaptive change specific to *A. halleri* could have been contemporary with the split from the *A. lyrata* lineage. This suggests that ecological differentiation may have occurred at the onset of speciation in this species pair. Similar approaches in the genus *Capsella* concluded to the co-occurrence of speciation in *C. rubella* with molecular changes at a locus strongly influencing plant fitness (the self-incompatibility locus, or S-locus, enforcing outcrossing in hermaphrodites) [32], [33]. This also occurred together with the evolution of a “selfing syndrome” in flower morphology, annual life cycle, and geographic expansion. Interestingly, similar features co-evolved very recently in *A. thaliana* [34], [35], [36], [37], but in clear disconnection with the time of split between *A. thaliana* and the lineage leading to its closely related species *A. halleri* and *A. lyrata*, which diverged much earlier [11], [38]. Hence, these contrasting patterns suggest that the shift in mating system from outcrossing to selfing may have been a key element of the speciation process in *C. rubella*, but not in *A. thaliana*.

The mechanisms by which divergent natural selection on phenotypic traits associated with ecological differentiation may promote reproductive isolation between populations are still largely unknown [39]. A key issue is to determine whether reproductive isolation associated with ecological speciation occurs mostly by direct or indirect effects of the adaptive molecular changes at target genes (2). In *A. halleri*, while increased expression of *AhHMA4* induced important changes in Zn translocation to aerial parts, the overall Zn tolerant phenotype results from a complex genetic architecture involving other genes of smaller effects [40]. Indeed, expression of *AhHMA4* in *A. thaliana* leads to elevated, rather than reduced, sensitivity to metals as a result of enhanced transfer from roots to shoots [15]. This negative effect of an *AhHMA4* transgene in an *A. thaliana* genomic background suggests that increased expression of *HMA4* in *A. halleri* necessitated the prior establishment of an adequate genetic background involving metal chelators, antioxydants, or metal transporters. This sequence of events is supported by the identification of several quantitative trait loci (QTL) regions involved in the tolerance to Zn and Cd in *A. halleri* [40]. In particular, one of these QTLs contains *MTP1* (*metal tolerance protein 1*), a gene involved in metal homeostasis [41] encoding a protein that mediates Zn transport from the cytoplasm to the vacuole [42], [43]. We propose that molecular changes at *HMA4* could have been favored in some appropriate genetic background characterized by preexisting *MTP1* mutants enabling plants to cope with elevated Zn in their aerial parts. Under this scenario, genetic exchanges between tolerant and non-tolerant populations would have generated low fitness genotypes, being hyperaccumulating yet highly sensitive, hence suggesting a direct involvement of the targets of adaptation in reproductive isolation.

Our suggestion that a major change in metal homeostasis would have occurred at the onset of *A. halleri* emergence is in line with the available data that indicates a species-wide pattern of strong Zn tolerance in *A. halleri* including populations from Western and Central Europe, Eastern Europe, Taiwan, and Japan [14], [44], [45]. However, the occurrence of species-wide metal tolerance long before the expansion of anthropogenic environments raises the issue of the ecological conditions that selected for this physiological change. An emerging hypothesis is the important role of metal hyperaccumulation in plant leaves as a defense mechanism against pathogens or herbivores [46], [47], [48], [49]. Alternatively, the natural occurrence of soils with high concentrations of Zn has been reported [50], but their restricted geographic distribution makes it difficult to understand how they could have played a major role, considering that the level of polymorphism observed in *A. halleri* precludes scenarios with a strong genetic bottleneck at speciation.

## Methods

### Plant material

For *A. halleri*, we sampled 31 diploid individuals from six populations scattered throughout the European distribution of the species [51]: F1, France (*N* = 6); I5, Italy (*N* = 5); D13, Germany (*N* = 5); SLO5, Slovenia (*N* = 5); PL1, Poland (*N* = 5); and CZ8, Czech Republic (*N* = 5). For *A. lyrata*, we used published sequences from four populations [17]: the Plech reference population in Germany (*N* = 12), which has been identified as part of the center of diversity of the species [17], [52], Sweden (*N* = 9), Iceland (*N* = 12) and Russia (*N* = 15).

### DNA sequencing

Large exons at 29 unlinked loci in *A. halleri* (Table S1) were amplified [30×(30″at 95°C, 45″ at 55°C, 60″ at 70°C)] using PCR primers defined for studies in *A. lyrata* [17], [53]. Restricting amplification within coding regions allowed us to perform direct sequencing, as it excluded the indels polymorphisms accumulating in intronic regions. PCR products were directly Sanger-sequenced using BigDye Terminator Kit 3.1 (Applied Biosystems, Foster City, CA). Chromatograms were checked manually using SeqScape V2.5. Included data were confirmed on both strands, and have been submitted to GenBank (accessions XXXXXX–XXXXXX).

### Data analysis

We used a routine written in C (MScalc, available upon request from xavier.vekemans@univ-lille1.fr) to compute diversity estimators at biallelic synonymous sites (nucleotide diversity π_s_; Watterson's θ_W_; F_ST_, computed as 1−π_s_/π_T_ where π_s_ is the average pairwise nucleotide diversity within population and π_T_ is the total pairwise nucleotide diversity of the pooled sample across populations). Seven different classes of polymorphic sites defined by Ramos-Onsins [21] were also computed, using sequences from the *A. thaliana* reference genome as outgroup : (1) exclusive polymorphisms noted *S*x_hal_ (or *S*x_lyr_), *i.e.* polymorphic sites for which the frequency of the derived allele f(*d*) is equal to 0 in *A. lyrata* (or in *A. halleri*) but 0<f(*d*)<1 in *A. halleri* (or *A. lyrata*); (2) fixed differences between species, noted *S*f_hal_ (or *S*f_lyr_), where f(*d*) = 1 in *A. halleri* and f(*d*) = 0 in *A. lyrata* (or vice versa); (3) shared polymorphic sites (noted *S*s), i.e. sites where 0<f(*d*)<1 in both species; and (4) exclusive polymorphisms that are fixed for the derived allele in the other species, noted *S*x_hal_f_lyr_ (or *S*x_lyr_f_hal_), *i.e.* f(*d*) = 1 in *A. lyrata* (or in *A. halleri*) but 0<f(*d*)<1 in *A. halleri* (or in *A. lyrata*). To better understand the demographic history of *A. halleri* and *A. lyrata*, haplotypes were estimated from the unphased data by use of the PHASE algorithm [54] implemented in DNAsp [55]. From the phased genotypes, we extracted the largest non-recombining sequences by use of the IMgc program [56]. The resulting set of non-recombining sequences was only used for the haplotypes analysis.

### Approximate Bayesian computation (ABC) analysis

#### Coalescent simulations

We generated distributions of 22 summary statistics (Table S6) under different demographic scenarios of divergence between two populations by coalescent-based simulations using the program msnsam [17], [57]. For each locus, coalescent simulations were performed based on corresponding sample sizes for *A. halleri* and *A. lyrata*, and based on the observed synonymous sequence length *L.* Mutations rates at all loci were estimated from the net nucleotide divergence at synonymous sites between *A. halleri* or *A. lyrata* and *A. thaliana*, assuming a divergence time of 5 MY [10] and an average generation time of two years (Table S7). Note that although the estimate for the divergence time with *A. thaliana* has been challenged recently [11], [58], our conclusions would not be altered since speciation times and duplication events were calibrated similarly. We approximated the recombination rate ρ*_i_* = θ*_i_*, as this corresponds to observations in *A. lyrata* [59], [60], as well as our own observations in *A. halleri*.

#### Demographic scenarios

We defined four classes of demographic scenarios as described in [61] (Fig. 3), classified according to the chronological patterns of gene exchange between populations. Within each class of scenarios, three alternative models were simulated assuming either constant population size, exponential population growth, or a bottleneck specific to *A. halleri* followed by exponential population growth. For each of the 12 resulting models, 5×10^6^ multilocus simulations were performed. We used large uniform prior distributions for all parameters, and used identical prior distributions for parameters common to all models. Prior distributions for *N_hal_* and *N_lyr_* were uniform on the interval 0–300,000, prior distribution for *N_A_* was uniform on the interval 0–1,000,000. Prior distributions for migration rates in both directions were uniform on the interval 0–20. We sampled *T_split_* from the interval 0–3,200,000 years. The parameters *T_iso_* and *T_SC_* were drawn from a uniform distribution on the interval 0-*T_split_*.

#### Procedure for model testing

For model testing, we followed a two-step hierarchical procedure [8]. First, for each class of scenarios, we evaluated posterior probabilities separately for the constant population size scenarios compared with either of the two alternative scenarios. Second, we compared the best models from the four classes of scenarios. Posterior probabilities for each candidate model were estimated using a feed-forward neural network implementing non-linear multivariate regression by considering the model itself as an additional parameter to be inferred under the ABC framework using the R package “abc” [62], [63], [64]. The 0.1% replicate simulations nearest to the observed values for the summary statistics (Table S6) were selected, and these were weighted by an Epanechnikov kernel that reaches a maximum when S_obs_ = S_sim_. Computations were performed by using 50 trained neural networks and 10 hidden networks in the regression. We described the test for the power of our model choice procedure in Text S1.

#### Procedure for parameter estimation

We estimated the posterior distributions of the parameters for the best model using a non-linear regression procedure. Parameters were first transformed according to a log-tangent transformation [65]. We considered only the 2,000 replicate simulations with the smallest associated Euclidean distance δ = ∥S_obs_−S_sim_∥. The joint posterior distribution of parameters describing the best model was obtained by means of weighted non-linear multivariate regressions of the parameters on the summary-statistics (Table S6). One hundred feed-forward neural networks and 15 hidden networks were trained for each regression using the R package “abc” [62] and results were averaged over the replicate networks. We performed a goodness of fit test with additional summary statistics on the results of parameter estimation to ensure that the estimated model fits the data as described in Text S1.

#### Estimation of *AhHMA4* duplication times

Complete coding sequences of the three copies of *AhHMA4* found in *A. halleri* were obtained from BAC sequences deposited in GenBank [15], [40]. The single copy of *AlHMA4* found on linkage group 3 in *A. lyrata* was obtained from the JGI database. The single copy found on chromosome 2 in *A. thaliana* was obtained from the TAIR database. The occurrence of gene conversion was assessed by using the program GENCONV [26]. Maximum-likelihood phylogenetic analyses were conducted in PhyML [66], [67], [68] using the best substitution model determined according to the software MODELTEST [69]. BEAST (v.1.5.3) [70] was used to date duplication events. The molecular clock model used was the relaxed, uncorrelated lognormal clock. The analyses performed on third codon positions were calibrated by using a normal prior on the age of the *A. thaliana*-[*A. halleri*/*A. lyrata*] divergence (median 5 MY, with 95% of the distribution lying between 4.5 and 5.5 MY [10]). A Yule process assuming a constant speciation rate per lineage was used for the speciation model. Posterior distributions were obtained by Markov chain Monte Carlo (MCMC) sampling, with 30,000 samples drawn from a total of 1×10^8^ steps, and a 3×10^7^ steps long burn-in. Quality of mixing and convergence to the stationary distribution were assessed from three independent runs by using Tracer v1.5 [70].

## Supporting Information

Text S1 Description of the different sampling strategies used in ABC analyzes and description of the methods for the model checking computation and the goodness-of-fit test.
(DOCX)

Figure S1 Composition of synonymous polymorphic sites (*A*) per locus and (*B*) across all loci, when all *A. lyrata* populations are pooled.
(TIF)

Figure S2 (*A*) Empirical distributions of the estimated relative probabilities of the SIC (black line), CMC (blue), AMC (green) and SCC (red) models when they are the true models. The area under each curve to the right of the vertical line represents the fraction of times that the true model is recovered (relative probability >0.5) by our estimation procedure, which amounts to 79.5% for the SIC, 90.8% for the CMC, 89.4% for the AMC, and 84.3% for the SCC. (*B*) Empirical distributions of the estimated relative probabilities of the SIC model when the SIC (black solid line), CMC (green dashed line), AMC (blue dashed line) or the SCC (red dashed line) models are the true models. The density estimates of the four models at the SIC posterior probability = 0.771 (vertical line) were used to compute the probability that SIC is the correct model given our observation that *P*_SIC_ = 0.771. This probability is equal to 0.975.
(TIF)

Figure S3 Posterior distributions for the parameters of the best population divergence model (SIC). Dashed curves represent the Bayesian prior for each parameter.
(TIF)

Figure S4 Phylogram representing preferred trees of orthologous and paralogous copies of *HMA4* in *Arabidopsis* computed with PhyML.
(TIF)

Table S1 Description of the loci surveyed giving their identification, chromosomal location, and annotation based on the A. thaliana genome.
(DOCX)

Table S2 Distribution of polymorphic sites into different categories of polymorphisms based on the pooled sample of all *A. lyrata* populations. See the Text S1 for a description of the categories (Methods: Data analysis).
(DOCX)

Table S3 Statistics of synonymous and non-synonymous diversity within *A. halleri* and *A. lyrata* species samples for each locus, and results from tests of the neutral hypothesis computed on synonymous sites.
(DOCX)

Table S4 Estimates of population nucleotide variation.
(DOCX)

Table S5 Posterior probabilities of SIC, CMC, AMC and SCC speciation models in four different analyses according to two sample schemes and the two sets of loci, either compared to SIE, CME, AME and SCE models or to SIB, CMB, AMB and SCB models.
(DOCX)

Table S6 Summary statistics used in the different procedures of the ABC analysis.
(DOCX)

Table S7 Results (*P*-values) of the goodness-of-fit tests for the SIC and AMC models for each of the four datasets.
(DOCX)

Table S8 Levels per locus of synonymous (K_syn_) and non-synonymous (K_asyn_) divergence among *Arabidopsis* species, estimates of F_ST_ per locus and mutation rates per bp per generation assuming a divergence time of 5 MY with *A. thaliana*.
(DOCX)
