# Supplementary material for: Does Speciation between Arabidopsis halleri and Arabidopsis lyrata Coincide with Major Changes in a Molecular Target of Adaptation?
Source: PLoS One. 2011 Nov 1;6(11):e26872. doi: 10.1371/journal.pone.0026872 (PMC3206069; doi:10.1371/journal.pone.0026872)
Supplement: Text S1 — Description of the different sampling strategies used in ABC analyzes and description of the methods for the model checking computation and the goodness-of-fit test. (DOCX) [file pone.0026872.s001.docx]

**Supporting Information Text**

**I. Robustness of ABC analyses in regard to the choice of loci and population samples from *A. lyrata***

To check the robustness of the analyses in regard to the sampling strategy in *A. lyrata* and to the choice of loci, we performed four ABC analyses (“Pool28”, “Plech28”, “Pool19”, and “Plech19”) with different numbers of loci (either 28 or 19) and different sample schemes for *A. lyrata* populations (“Pool” and “Plech” analyses):

**A. Selection of loci.** ABC analyses were performed in two different ways according to the number of loci used in demographic inferences. In the first set, 28 loci were included, i.e. all 29 sequenced loci except *At1g72390*, which was monomorphic in both species; the second set contained 19 loci after nine loci (*At1g01040, At1g15240, At1g59720, At1g62390, At1g74600, At2g26140, At3g48690, At3g50740* and *At3g62890*) were removed from the analysis because they produced significant neutrality tests for all three applied statistics: Tajima’s *D* [1], Fu’s *Fs* [2] and Ramos-Onsins & Rozas *R2* [3], or because HKA tests [4] showed significant departure from neutrality. As shown in Table S3, the *D*, *F_S_*, and *R_2_* statistics were computed on synonymous positions to test the neutral evolution hypothesis in both populations. Observed values were compared to simulated distributions obtained from 3,000 coalescent simulations under the neutral null hypothesis using MANVa (http://www.ub.edu/softevol/manva). A maximum-likelihood multilocus test of the standard neutral model based on the Hudson-Kreitman-Aguade test was applied to polymorphism data for the 29 genes from *A. halleri* and *A. lyrata* using divergence data from *A. thaliana* [5].

**B. *A. lyrata* sample schemes.** To ensure that the previously reported population structure across the four studied *A. lyrata* populations [6] would not severely affect our demographic conclusions [7], we performed ABC analyses under two alternative sample schemes: “Pool” sample where all *A. lyrata* populations were pooled together; “Plech” sample where only the German population was used because it has been identified as part of the center of diversity of this species [6].

The results from the model choice procedure were very similar for all four analyses (Table S5): in each class of scenarios, the model with constant population size produced higher posterior probability than the two alternative models; in comparisons among the best models from each class of scenarios, the strict isolation scenario (SI) produced higher posterior probability than the three alternative models, with the exception of the Plech19 analysis which generated very similar posterior probabilities under the strict isolation and ancient migration scenarios.

**II. Robustness of the model choice procedure**

To test the robustness of the results from the model choice procedure in the ABC analysis, we analyzed 1,000 datasets simulated for each “constant population size” version of the SI, CM, AM, and SC models, obtained by drawing random parameter values from the same prior distribution as the analysis performed on the observed dataset.

We first estimated the robustness of the model choice procedure within each class of scenarios (SI, CM, AM and SC) in regard to pairwise comparisons of the model with constant population size with either the exponential population growth model or the recent bottleneck in *A. halleri* model. For each simulated dataset, and each pairwise comparison of models, we applied the model choice procedure of the ABC analysis to compute the posterior probabilities of the two models under comparison. Following [8], the distributions of these probabilities over the 1,000 replicates were then used to compute the probabilities for each class of scenarios that the constant population model was the correct model (compared to either of the two alternative models) given the posterior probability obtained for the observed dataset (values in parentheses in Table 1). The results showed that for all classes of scenarios, the constant population size model had the highest probability of being the correct model, with most probability values greater than 0.9.

Given the strong support for the constant population size model, we estimated the robustness of the model choice procedure among the four classes of scenarios. First, we checked whether the correct scenario was accurately picked by the model selection procedure when applied to simulated datasets. From the previous 1,000 simulated datasets performed on each scenario, we estimated the distributions of the posterior probability of the correct model (Fig. S2*A*). The proportion of simulated data sets correctly recovered with a posterior probability of >0.5 by our estimation procedure was 79.5% for SIC, 90.8% for CMC, 89.4% for AMC, and 84.3% for SCC (Fig. S2*A*). Therefore, since the SIC model was consistently the most difficult model to identify correctly, our results (that the SIC has a higher posterior probability based on the observed dataset) cannot be attributed to a false positive. Based on the 1,000 simulated datasets according to each of the four models, we computed the relative probability that SIC is the correct model given the observation that *P_SIC_*=0.771 as Pr(*P_SIC_*=0.771 | SIC)/ [Pr(*P_SIC_*=0.771 | SIC)+ Pr(*P_SIC_*=0.771 | CMC)+ Pr(*P_SIC_*=0.771 | AMC)+ Pr(*P_SIC_*=0.771 | SCC)]=0.975 (Figure S2-B).

**III. Goodness of fit**

For the SIC and AMC models, we tested whether the parameter values drawn from the posterior distribution estimated by our ABC approach fit the data using a goodness-of-fit test. First, 2,000 multilocus datasets were simulated from the posterior distribution of parameter values using a modified version of ms, msnsam, which can simulate different sample sizes across loci. From these simulations, we computed the distributions of 34 summary statistics (Table S5) under the estimated model as described in [9]. These distributions can be used to calculate the predictive p-value as the probability of obtaining the observed value of a statistic or a more extreme value under the inferred scenario. A model fits the data correctly when the calculated p-value for each summary statistic is higher than 0.05. As shown in Table S6, the 34 *P*-values obtained range from 0.042 to 0.478, with a single statistic associated with a p-value smaller than 0.05, i.e. the statistic related to the standard variation of S_x_*_hal_*_-f_*_lyr_* . Hence, these results suggest that our best model generally fit the data well under this criterion.

1. Tajima F (1989) Statistical Method for Testing the Neutral Mutation Hypothesis by DNA Polymorphism. Genetics 123: 585-595.

2. Fu YX (1996) New Statistical Tests of Neutrality for DNA Samples From a Population. Genetics 143: 557-570.

3. Ramos-Onsins SE, Rozas J (2002) Statistical Properties of New Neutrality Tests Against Population Growth. Mol Biol Evol 19: 2092-2100.

4. Hudson RR, Kreitman M, Aquade M (1987) A test of neutral molecular evolution based on nucleotide data. Genetics 116: 153-159.

5. Wright SI, Charlesworth B (2004) The HKA Test Revisited: A Maximum-Likelihood-Ratio Test of the Standard Neutral Model. Genetics 168: 1071-1076.

6. Ross-Ibarra J, Wright SI, Foxe JP, Kawabe A, DeRose-Wilson L, et al. (2008) Patterns of Polymorphism and Demographic History in Natural Populations of <italic>Arabidopsis lyrata</italic>. PLoS ONE 3: e2411.

7. Stadler T, Haubold B, Merino C, Stephan W, Pfaffelhuber P (2009) The Impact of Sampling Schemes on the Site Frequency Spectrum in Nonequilibrium Subdivided Populations. Genetics 182: 205-216.

8. Fagundes NJR, Ray N, Beaumont M, Neuenschwander S, Salzano FM, et al. (2007) Statistical evaluation of alternative models of human evolution. Proceedings of the National Academy of Sciences 104: 17614-17619.

9. Becquet C, Przeworski M (2009) Learning about modes of speciation by computational approaches. Evolution 63: 2547-2562.
