# Supplementary figures and images for: Does Speciation between Arabidopsis halleri and Arabidopsis lyrata Coincide with Major Changes in a Molecular Target of Adaptation?
Source: PLoS One. 2011 Nov 1;6(11):e26872. doi: 10.1371/journal.pone.0026872 (PMC3206069; doi:10.1371/journal.pone.0026872)

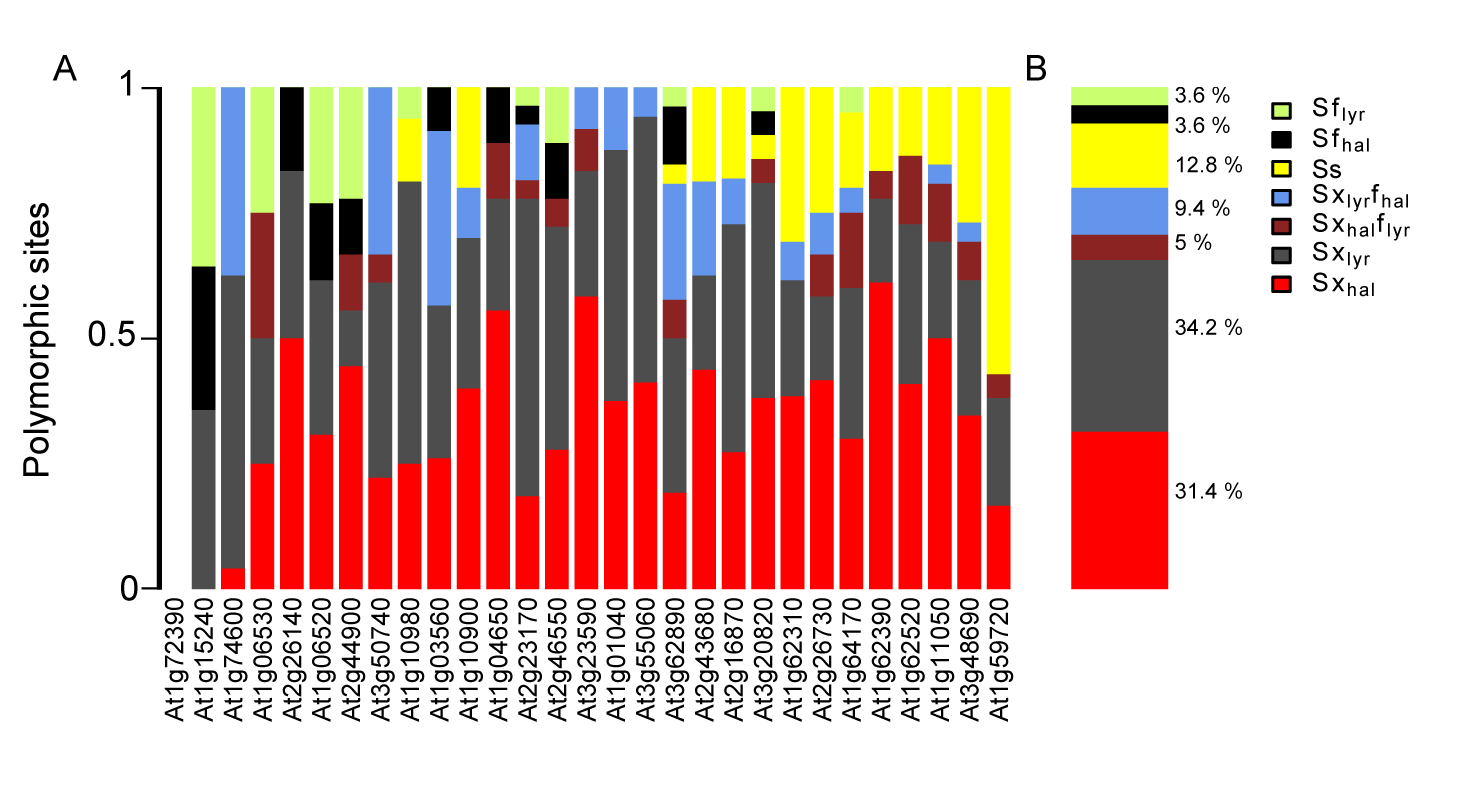

Supplement: Figure S1 — Composition of synonymous polymorphic sites (A) per locus and (B) across all loci, when all A. lyrata populations are pooled. (TIF) [file pone.0026872.s002.tif]

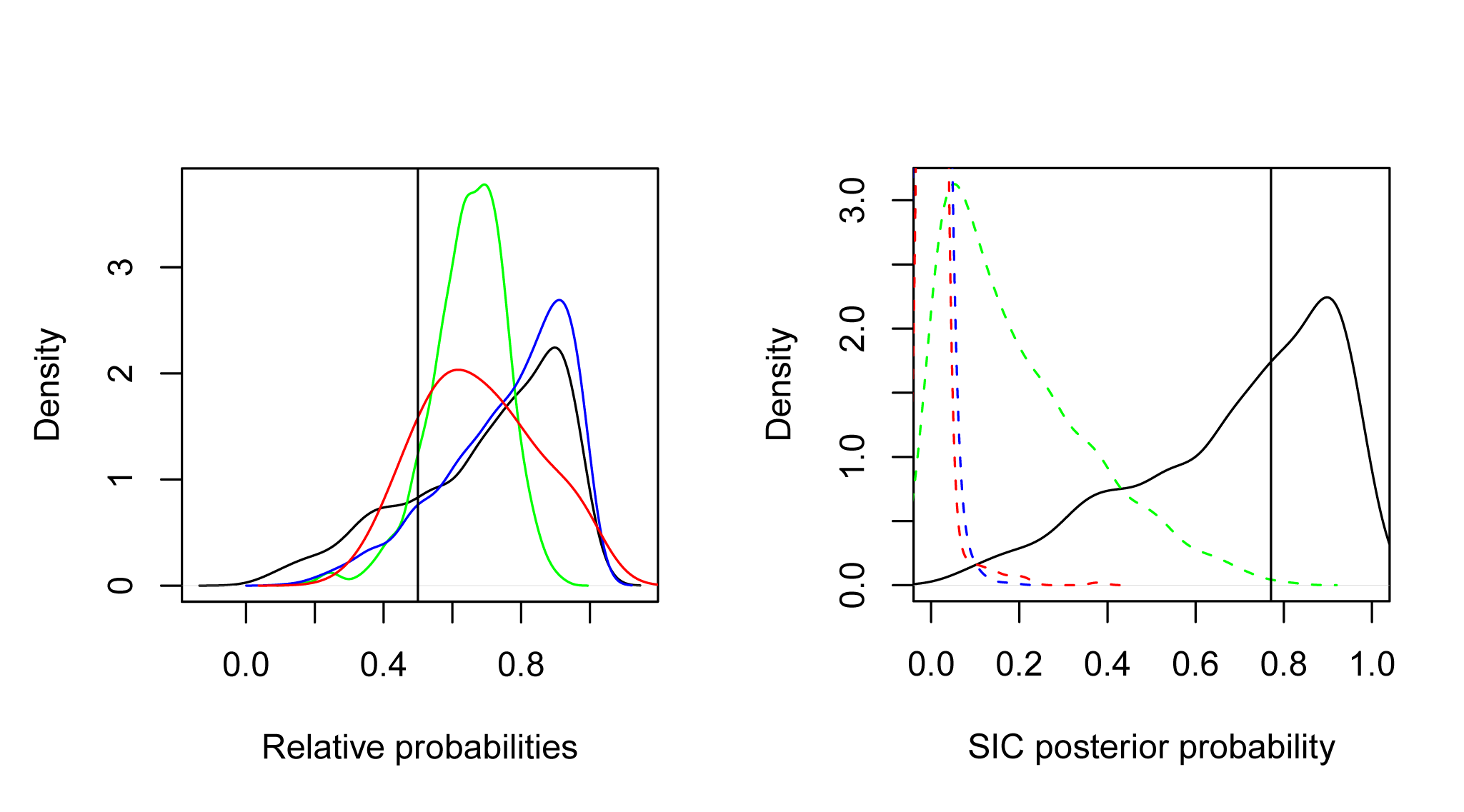

Supplement: Figure S2 — (A) Empirical distributions of the estimated relative probabilities of the SIC (black line), CMC (blue), AMC (green) and SCC (red) models when they are the true models. The area under each curve to the right of the vertical line represents the fraction of times that the true model is recovered (relative probability >0.5) by our estimation procedure, which amounts to 79.5% for the SIC, 90.8% for the CMC, 89.4% for the AMC, and 84.3% for the SCC. (B) Empirical distributions of the estimated relative probabilities of the SIC model when the SIC (black solid line), CMC (green dashed line), AMC (blue dashed line) or the SCC (red dashed line) models are the true models. The density estimates of the four models at the SIC posterior probability = 0.771 (vertical line) were used to compute the probability that SIC is the correct model given our observation that P SIC = 0.771. This probability is equal to 0.975. (TIF) [file pone.0026872.s003.tif]

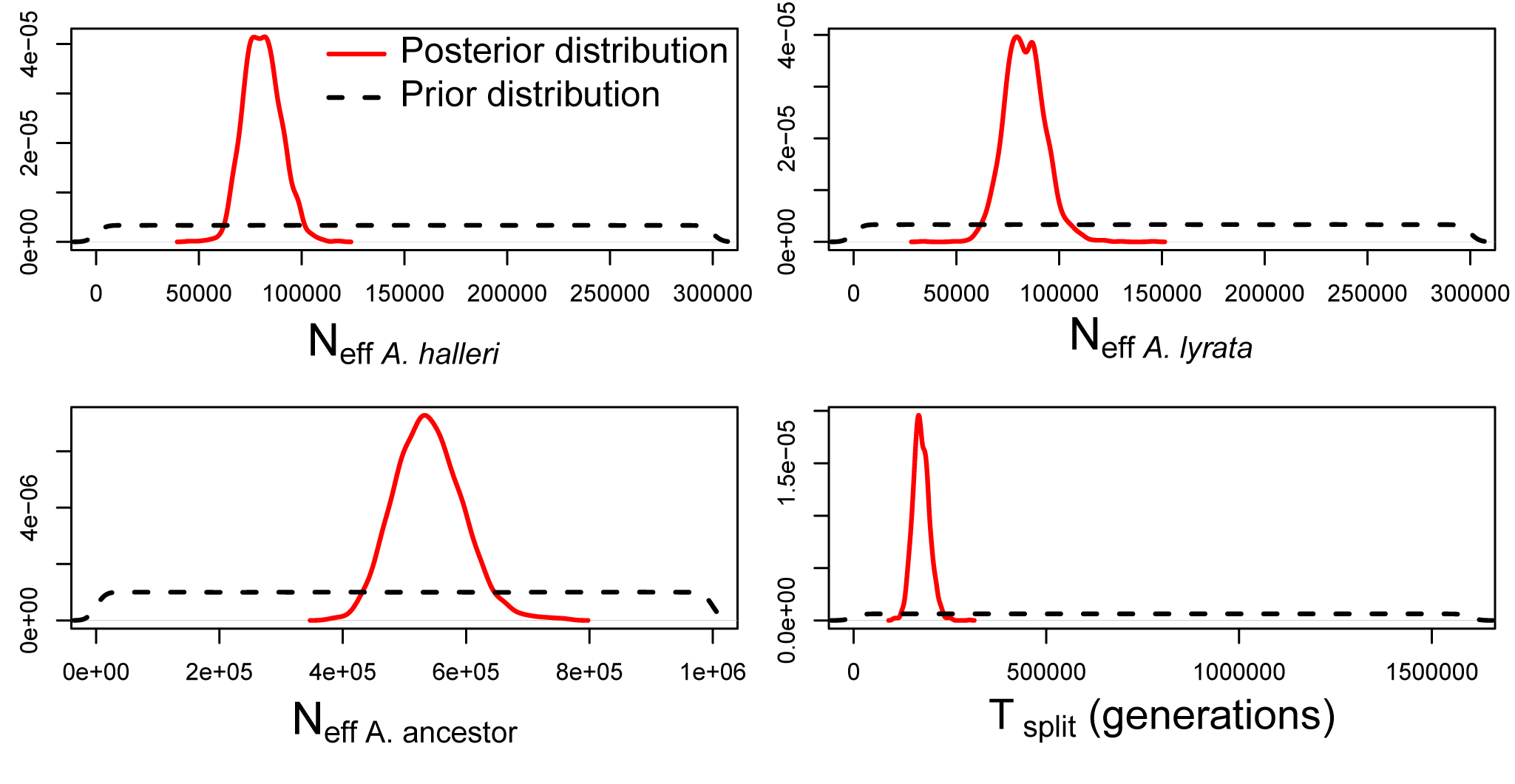

Supplement: Figure S3 — Posterior distributions for the parameters of the best population divergence model (SIC). Dashed curves represent the Bayesian prior for each parameter. (TIF) [file pone.0026872.s004.tif]

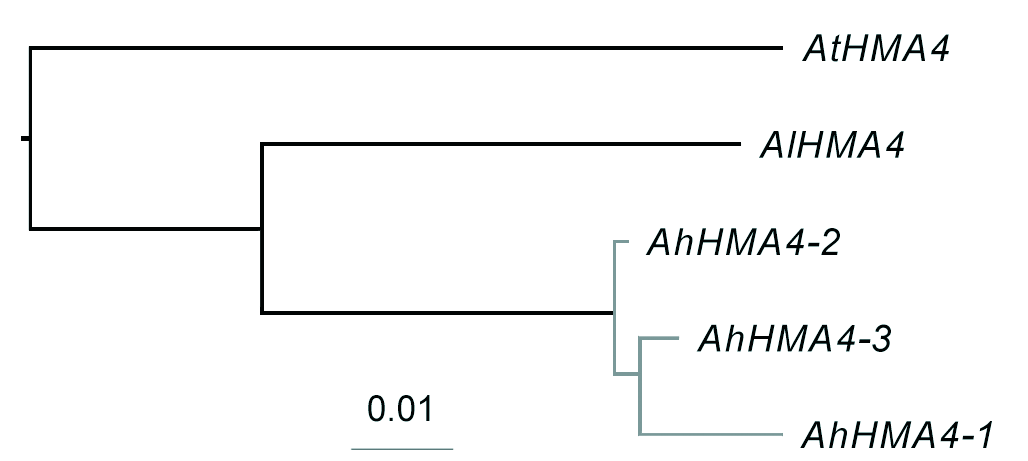

Supplement: Figure S4 — Phylogram representing preferred trees of orthologous and paralogous copies of HMA4 in Arabidopsis computed with PhyML. (TIF) [file pone.0026872.s005.tif]
