# Supplementary material for: Does Speciation between Arabidopsis halleri and Arabidopsis lyrata Coincide with Major Changes in a Molecular Target of Adaptation?
Source: PLoS One. 2011 Nov 1;6(11):e26872. doi: 10.1371/journal.pone.0026872 (PMC3206069; doi:10.1371/journal.pone.0026872)
Supplement: Table S1 — Description of the loci surveyed giving their identification, chromosomal location, and annotation based on the A. thaliana genome. (DOCX) [file pone.0026872.s006.docx]

| **Gene** | **Number of Sites (bp)** | | | GO Terms |
| --- | --- | --- | --- | --- |
|  | **Total** | **Synonymous** | **Non-Synonymous** |  |
| *At1g01040* | 437 | 96 | 341 | DCL-1 |
| *At1g03560* | 484 | 110 | 374 | unknown protein |
| *At1g04650* | 458 | 108 | 351 | unknown protein |
| *At1g06520* | 448 | 101 | 346 | Acyltransferase activity. Expressed in flower buds and siliques. |
| *At1g06530* | 447 | 86 | 361 | unknown protein |
| *At1g10900* | 481 | 110 | 371 | 1-phosphatidylinositol-4-phosphate 5-kinase |
| *At1g10980* | 495 | 113 | 382 | unknown protein |
| *At1g11050* | 468 | 115 | 353 | Kinase activity |
| *At1g15240* | 426 | 91 | 338 | unknown protein |
| *At1g59720* | 483 | 109 | 374 | unknown protein |
| *At1g62310* | 458 | 94 | 362 | Transcription factor jumonji (jmjC) |
| *At1g62390* | 483 | 99 | 384 | unknown protein |
| *At1g62520* | 423 | 99 | 325 | unknown protein |
| *At1g64170* | 423 | 110 | 313 | unknown protein |
| *At1g72390* | 371 | 78 | 293 | unknown protein |
| *At1g74600* | 513 | 120 | 393 | unknown protein |
| *At2g16870* | 544 | 121 | 422 | Disease resistance protein (TIR-NBS-LRR class) |
| *At2g23170* | 451 | 108 | 340 | IAA-amido synthase |
| *At2g26140* | 435 | 100 | 335 | FtsH protease |
| *At2g26730* | 349 | 86 | 263 | Leucine-rich repeat transmembrane protein kinase |
| *At2g43680* | 514 | 120 | 386 | calmodulin-binding family protein, similar to SF16 |
| *At2g44900* | 447 | 108 | 339 | ubiquitin-protein ligase activity |
| *At2g46550* | 437 | 99 | 331 | unknown protein |
| *At3g20820* | 491 | 124 | 365 | unknown protein |
| *At3g23590* | 523 | 132 | 390 | unknown protein |
| *At3g48690* | 447 | 91 | 353 | Similar to PrMC3 |
| *At3g50740* | 444 | 107 | 337 | DP-glucoronosyl/UDP-glucosyl transferase family |
| *At3g55060* | 465 | 96 | 369 | unknown protein |
| *At3g62890* | 453 | 104 | 350 | unknown protein |
| Total | 13,298 | 3,035 | 10,241 |  |
