# Supplementary material for: Does Speciation between Arabidopsis halleri and Arabidopsis lyrata Coincide with Major Changes in a Molecular Target of Adaptation?
Source: PLoS One. 2011 Nov 1;6(11):e26872. doi: 10.1371/journal.pone.0026872 (PMC3206069; doi:10.1371/journal.pone.0026872)
Supplement: Table S2 — Distribution of polymorphic sites into different categories of polymorphisms based on the pooled sample of all A. lyrata populations. See the Text S1 for a description of the categories (Methods: Data analysis). (DOCX) [file pone.0026872.s007.docx]

| Locus | Bp_syn_ | Bp_asyn_ | Sx*_hal_*_-syn_ | Sx*_hal_*_-asyn_ | Sx*_lyr_*_-syn_ | Sx*_lyr_*_-asyn_ | Sf*_hal_*_-syn_ | Sf*_hal_*_-asyn_ | Sf*_lyr_*_-syn_ | Sf*_lyr_*_-asyn_ | Sx*_hal_*f*_lyr_*_-syn_ | Sx*_hal_*f*_lyr_*_-asyn_ | Sx*_lyr_*f*_hal_*_-syn_ | Sx*_lyr_*f*_hal_*_-asyn_ | Ss_syn_ | Ss_asyn_ |
| --- | --- | --- | --- | --- | --- | --- | --- | --- | --- | --- | --- | --- | --- | --- | --- | --- |
| *At1g01040* | 96 | 341 | 6 | 4 | 8 | 3 | 0 | 0 | 0 | 0 | 0 | 1 | 2 | 0 | 0 | 0 |
| *At1g03560* | 110 | 374 | 6 | 4 | 7 | 7 | 2 | 0 | 0 | 0 | 0 | 0 | 8 | 2 | 0 | 1 |
| *At1g04650* | 108 | 351 | 5 | 8 | 2 | 6 | 1 | 0 | 0 | 1 | 1 | 2 | 0 | 0 | 0 | 0 |
| *At1g06520* | 101 | 346 | 4 | 4 | 4 | 4 | 2 | 0 | 3 | 1 | 0 | 0 | 0 | 0 | 0 | 0 |
| *At1g06530* | 86 | 361 | 1 | 5 | 1 | 2 | 0 | 0 | 1 | 1 | 1 | 1 | 0 | 0 | 0 | 0 |
| *At1g10900* | 110 | 371 | 4 | 4 | 3 | 7 | 0 | 0 | 0 | 0 | 0 | 0 | 1 | 1 | 2 | 1 |
| *At1g10980* | 113 | 382 | 4 | 8 | 9 | 14 | 0 | 2 | 1 | 0 | 0 | 0 | 0 | 1 | 2 | 0 |
| *At1g11050* | 115 | 353 | 13 | 2 | 5 | 5 | 0 | 1 | 0 | 0 | 3 | 3 | 1 | 1 | 4 | 0 |
| *At1g15240* | 91 | 338 | 0 | 3 | 5 | 5 | 4 | 5 | 5 | 2 | 0 | 0 | 0 | 0 | 0 | 0 |
| *At1g59720* | 109 | 374 | 7 | 13 | 9 | 10 | 0 | 0 | 0 | 0 | 2 | 1 | 0 | 0 | 24 | 11 |
| *At1g62310* | 94 | 362 | 5 | 6 | 3 | 10 | 0 | 0 | 0 | 0 | 0 | 1 | 1 | 0 | 4 | 1 |
| *At1g62390* | 99 | 384 | 11 | 2 | 3 | 4 | 0 | 0 | 0 | 0 | 1 | 1 | 0 | 1 | 3 | 0 |
| *At1g62520* | 99 | 325 | 9 | 4 | 7 | 1 | 0 | 0 | 0 | 1 | 3 | 0 | 0 | 0 | 3 | 0 |
| *At1g64170* | 110 | 313 | 6 | 4 | 6 | 2 | 0 | 0 | 1 | 0 | 3 | 1 | 1 | 0 | 3 | 0 |
| *At1g72390* | 78 | 293 | 0 | 3 | 0 | 1 | 0 | 0 | 0 | 1 | 0 | 0 | 0 | 0 | 0 | 0 |
| *At1g74600* | 120 | 393 | 1 | 2 | 14 | 5 | 0 | 0 | 0 | 0 | 0 | 0 | 9 | 1 | 0 | 3 |
| *At2g16870* | 121 | 422 | 6 | 10 | 10 | 13 | 0 | 0 | 0 | 1 | 0 | 2 | 2 | 0 | 4 | 3 |
| *At2g23170* | 108 | 340 | 5 | 0 | 16 | 3 | 1 | 1 | 1 | 1 | 1 | 0 | 3 | 0 | 0 | 0 |
| *At2g26140* | 100 | 335 | 3 | 0 | 2 | 0 | 1 | 0 | 0 | 0 | 0 | 0 | 0 | 0 | 0 | 0 |
| *At2g26730* | 86 | 263 | 5 | 0 | 2 | 0 | 0 | 0 | 0 | 0 | 1 | 0 | 1 | 0 | 3 | 0 |
| *At2g43680* | 120 | 386 | 7 | 7 | 3 | 3 | 0 | 0 | 0 | 0 | 0 | 0 | 3 | 0 | 3 | 1 |
| *At2g44900* | 108 | 339 | 4 | 7 | 1 | 2 | 1 | 1 | 2 | 0 | 1 | 0 | 0 | 0 | 0 | 0 |
| *At2g46550* | 99 | 331 | 5 | 8 | 8 | 3 | 2 | 2 | 2 | 0 | 1 | 0 | 0 | 0 | 0 | 0 |
| *At3g20820* | 124 | 365 | 8 | 1 | 9 | 5 | 1 | 0 | 1 | 0 | 1 | 0 | 0 | 0 | 1 | 0 |
| *At3g23590* | 132 | 390 | 7 | 5 | 3 | 6 | 0 | 0 | 0 | 1 | 1 | 1 | 1 | 0 | 0 | 0 |
| *At3g48690* | 91 | 353 | 9 | 6 | 7 | 1 | 0 | 0 | 0 | 3 | 2 | 1 | 1 | 0 | 7 | 2 |
| *At3g50740* | 107 | 337 | 4 | 5 | 7 | 5 | 0 | 0 | 0 | 0 | 1 | 0 | 6 | 0 | 0 | 0 |
| *At3g55060* | 96 | 369 | 7 | 8 | 9 | 3 | 0 | 0 | 0 | 1 | 0 | 0 | 1 | 0 | 0 | 0 |
| *At3g62890* | 104 | 350 | 5 | 2 | 8 | 7 | 3 | 3 | 1 | 0 | 2 | 1 | 6 | 3 | 1 | 0 |
| Total | 3,035 | 10,241 | 157 | 135 | 171 | 137 | 18 | 15 | 18 | 14 | 25 | 16 | 47 | 10 | 64 | 23 |
