# Supplementary material for: Does Speciation between Arabidopsis halleri and Arabidopsis lyrata Coincide with Major Changes in a Molecular Target of Adaptation?
Source: PLoS One. 2011 Nov 1;6(11):e26872. doi: 10.1371/journal.pone.0026872 (PMC3206069; doi:10.1371/journal.pone.0026872)
Supplement: Table S3 — Statistics of synonymous and non-synonymous diversity within A. halleri and A. lyrata species samples for each locus, and results from tests of the neutral hypothesis computed on synonymous sites. (DOCX) [file pone.0026872.s008.docx]

| **Locus** | **Species** | **n samp** | **π_syn_** | **π_asyn_** | **π_asyn_/π_syn_** | **θ_syn_** | **θ_asyn_** | **θ_asyn_/θ_syn_** | **Tajima's *D*** | **Fu's *Fs*** | ***R2*** | ***K*(MLHKA)** |
| --- | --- | --- | --- | --- | --- | --- | --- | --- | --- | --- | --- | --- |
| *At1g01040* | *A.halleri* | 56 | 0.0199 | 0 | 0.05 | 0.013 | 0 | 0.237 | 1.184* | 1.963* | 0.185* | 1.037 |
|  | *A.lyrata (Plech)* | 18 | 0.033 | 0 | 0.061 | 0.018 | 0 | 0.047 | 2.598*** | 6.399*** | 0.264*** | 1.171 |
|  | *A.lyrata (Pool)* | 72 | 0.026 | 0 | 0.114 | 0.021 | 0 | 0.085 | 0.648 | NA | NA | NA |
| *At1g03560* | *A.halleri* | 56 | 0.0128 | 0 | 0.157 | 0.012 | 0 | 0.247 | 0.209 | 1.991* | 0.156 | 0.48 |
|  | *A.lyrata (Plech)* | 22 | 0.045 | 0.01 | 0.133 | 0.037 | 0.01 | 0.178 | 0.781 | 5.595 | 0.165 | 2.171 |
|  | *A.lyrata (Pool)* | 84 | 0.061 | 0.01 | 0.132 | 0.027 | 0.01 | 0.197 | 3.515 | NA | NA | NA |
| *At1g04650* | *A.halleri* | 58 | 0.0051 | 0 | 0.586 | 0.012 | 0.01 | 0.516 | -1.407 | -1.57 | 0.048* | 0.975 |
|  | *A.lyrata (Plech)* | 18 | 0.002 | 0 | 1 | 0.005 | 0 | 0.619 | -1.508 | -1.744* | 0.157 | 0.525 |
|  | *A.lyrata (Pool)* | 84 | 4.00E-04 | 0 | 4.578 | 0.004 | 0 | 0.942 | -1.401 | NA | NA | NA |
| *At1g06520* | *A.halleri* | 62 | 0.0068 | 0 | 0.148 | 0.008 | 0 | 0.294 | -0.411 | -0.938 | 0.086 | 0.451 |
|  | *A.lyrata (Plech)* | 12 | 0.007 | 0 | 0.143 | 0.01 | 0 | 0.294 | -0.829 | -1.256 | 0.142 | 0.366 |
|  | *A.lyrata (Pool)* | 68 | 0.005 | 0 | 0.398 | 0.008 | 0 | 0.297 | -0.821 | NA | NA | NA |
| *At1g06530* | *A.halleri* | 60 | 0.011 | 0 | 0.184 | 0.005 | 0 | 0.713 | 2.017* | 0.847 | 0.233* | 0.329 |
|  | *A.lyrata (Plech)* | 22 | 0.002 | 0 | 1 | 0.003 | 0 | 0.475 | -0.641 | -0.176 | 0.086* | 0.23 |
|  | *A.lyrata (Pool)* | 44 | 0.004 | 0 | 0.239 | 0.003 | 0 | 0.476 | 0.783 | NA | NA | NA |
| *At1g10900* | *A.halleri* | 56 | 0.01 | 0 | 0.198 | 0.012 | 0 | 0.246 | -0.377 | -2.413 | 0.092 | 1.091 |
|  | *A.lyrata (Plech)* | 24 | 0.021 | 0 | 0.19 | 0.015 | 0.01 | 0.345 | 1.391 | 0.375 | 0.202 | 1.274 |
|  | *A.lyrata (Pool)* | 80 | 0.02 | 0 | 0.148 | 0.011 | 0.01 | 0.446 | 1.959 | NA | NA | NA |
| *At1g10980* | *A.halleri* | 52 | 0.007 | 0 | 0.46 | 0.012 | 0.01 | 0.393 | -1.124 | -1.363 | 0.078 | 1.576 |
|  | *A.lyrata (Plech)* | 16 | 0.024 | 0.01 | 0.208 | 0.021 | 0.01 | 0.332 | 0.446 | -2.493 | 0.159 | 1.76 |
|  | *A.lyrata (Pool)* | 76 | 0.012 | 0 | 0.32 | 0.02 | 0.01 | 0.399 | -1.007 | NA | NA | NA |
| *At1g11050* | *A.halleri* | 60 | 0.041 | 0 | 0.074 | 0.037 | 0 | 0.082 | 0.306 | 0.717 | 0.109 | 1.614 |
|  | *A.lyrata (Plech)* | 22 | 0.002 | 0 | 0.5 | 0.005 | 0 | 0.164 | -1.515 | -1.974 | 0.144 | 0.207 |
|  | *A.lyrata (Pool)* | 78 | 0.004 | 0 | 0.556 | 0.017 | 0 | 0.198 | -2.108 | NA | NA | NA |
| *At1g15240* | *A.halleri* | 62 | 0 | 0 | NA | 0 | 0 | NA | 0.000 | NA | NA | 0* |
|  | *A.lyrata (Plech)* | 20 | 0.013 | 0 | 0.077 | 0.012 | 0 | 0.136 | 0.820 | 0.225 | 0.184 | 0.548 |
|  | *A.lyrata (Pool)* | 40 | 0.02 | 0.01 | 0.297 | 0.013 | 0 | 0.268 | 1.477 | NA | NA | NA |
| *At1g59720* | *A.halleri* | 52 | 0.087 | 0.02 | 0.172 | 0.067 | 0.02 | 0.22 | 1.003 | -6.896 | 0.142 | 9.261* |
|  | *A.lyrata (Plech)* | 14 | 0.087 | 0.01 | 0.138 | 0.081 | 0.01 | 0.125 | 0.252 | 2.18** | 0.159 | 13.747* |
|  | *A.lyrata (Pool)* | 54 | 0.08 | 0.01 | 0.15 | 0.066 | 0.01 | 0.185 | 0.683 | NA | NA | NA |
| *At1g62310* | *A.halleri* | 52 | 0.029 | 0 | 0.07 | 0.021 | 0.01 | 0.233 | 1.025 | 1.327 | 0.141 | 1.708 |
|  | *A.lyrata (Plech)* | 16 | 0.02 | 0.01 | 0.25 | 0.025 | 0.01 | 0.262 | -0.294 | -0.781 | 0.129 | 2.108 |
|  | *A.lyrata (Pool)* | 76 | 0.005 | 0 | 0.56 | 0.017 | 0.01 | 0.357 | -1.744 | NA | NA | NA |
| *At1g62390* | *A.halleri* | 58 | 0.064 | 0 | 0.031 | 0.032 | 0 | 0.052 | 2.923*** | 8.831** | 0.213** | 2.643* |
|  | *A.lyrata (Plech)* | 18 | 0.025 | 0 | 0.12 | 0.017 | 0 | 0.174 | 1.494 | 2.349 | 0.211 | 2.117 |
|  | *A.lyrata (Pool)* | 70 | 0.02 | 0 | 0.102 | 0.012 | 0 | 0.215 | 1.358 | NA | NA | NA |
| *At1g62520* | *A.halleri* | 60 | 0.042 | 0 | 0.047 | 0.032 | 0 | 0.072 | 0.906 | -1.161 | 0.158 | 1.411 |
|  | *A.lyrata (Plech)* | 14 | 0.024 | 0 | 0 | 0.019 | 0 | 0 | 0.947 | 0.517 | 0.199 | 0.679 |
|  | *A.lyrata (Pool)* | 70 | 0.026 | 0 | 0 | 0.021 | 0 | 0.031 | 0.690 | NA | NA | NA |
| *At1g64170* | *A.halleri* | 56 | 0.028 | 0 | 0.071 | 0.024 | 0 | 0.148 | 0.586 | -1.879 | 0.131 | 2.113 |
|  | *A.lyrata (Plech)* | 20 | 0.012 | 0 | 0.083 | 0.018 | 0 | 0.051 | -1.168 | -0.124 | 0.11 | 1.06 |
|  | *A.lyrata (Pool)* | 76 | 0.027 | 0 | 0.075 | 0.018 | 0 | 0.07 | 1.178 | NA | NA | NA |
| *At1g72390* | *A.halleri* | 52 | 0 | 0 | NA | 0 | 0 | NA | NA | NA | NA | 0^*^ |
|  | *A.lyrata (Plech)* | 20 | 0 | 0 | NA | 0 | 0 | NA | NA | NA | NA | 0^*^ |
|  | *A.lyrata (Pool)* | 82 | 0 | 0 | NA | 0 | 0 | NA | NA | NA | NA | NA |
| *At1g74600* | *A.halleri* | 56 | 3.00E-04 | 0 | 10.081 | 0.002 | 0 | 1.523 | -1.091 | -1.731 | 0.132 | 0.069* |
|  | *A.lyrata (Plech)* | 22 | 0.088 | 0.01 | 0.125 | 0.048 | 0.01 | 0.131 | 3.111*** | 14.062*** | 0.252*** | 2.157 |
|  | *A.lyrata (Pool)* | 72 | 0.041 | 0.01 | 0.147 | 0.04 | 0.01 | 0.119 | 0.098 | NA | NA | NA |
| *At2g16870* | *A.halleri* | 56 | 0.021 | 0.01 | 0.426 | 0.018 | 0.01 | 0.463 | 0.519 | -2.486 | 0.133 | 1.006 |
|  | *A.lyrata (Plech)* | 18 | 0.033 | 0.01 | 0.333 | 0.026 | 0.01 | 0.315 | 0.827 | 0.279 | 0.176 | 1.738 |
|  | *A.lyrata (Pool)* | 52 | 0.037 | 0.01 | 0.244 | 0.029 | 0.01 | 0.287 | 0.831 | NA | NA | NA |
| *At2g23170* | *A.halleri* | 54 | 0.005 | 0 | 0 | 0.012 | 0 | 0 | -1.557* | -1.941 | 0.047* | 0.837 |
|  | *A.lyrata (Plech)* | 22 | 0.047 | 0 | 0.021 | 0.033 | 0 | 0.049 | 1.376* | -0.827 | 0.191 | 1.39 |
|  | *A.lyrata (Pool)* | 84 | 0.054 | 0 | 0.037 | 0.035 | 0 | 0.05 | 1.561 | NA | NA | NA |
| *At2g26140* | *A.halleri* | 62 | 0.006 | 0 | 0 | 0.006 | 0 | 0 | -0.029 | -0.143 | 0.108 | 0.354 |
|  | *A.lyrata (Plech)* | 22 | 0 | 0 | NA | 0 | 0 | NA | NA | NA | NA | 0^*^ |
|  | *A.lyrata (Pool)* | 74 | 0.003 | NA | NA | 0.004 | NA | NA | -0.431 | NA | NA | NA |
| *At2g26730* | *A.halleri* | 62 | 0.032 | 0 | 0 | 0.022 | 0 | 0 | 1.252 | 4.06* | 0.155 | 0.725 |
|  | *A.lyrata (Plech)* | 18 | 0.005 | 0 | 0 | 0.013 | 0 | 0 | -1.853* | 1.116 | 0.229* | 0.497 |
|  | *A.lyrata (Pool)* | 32 | 0.013 | NA | NA | 0.017 | NA | NA | -0.703 | NA | NA | NA |
| *At2g43680* | *A.halleri* | 62 | 0.009 | 0 | 0.334 | 0.018 | 0 | 0.25 | -1.349 | -7.262 | 0.058 | 0.853 |
|  | *A.lyrata (Plech)* | 10 | 0.02 | 0 | 0.1 | 0.02 | 0 | 0.13 | -0.382 | 1.176** | 0.187 | 1.435 |
|  | *A.lyrata (Pool)* | 50 | 0.031 | 0 | 0.128 | 0.017 | 0 | 0.136 | 2.470 | NA | NA | NA |
| *At2g44900* | *A.halleri* | 60 | 0.008 | 0 | 0.367 | 0.01 | 0 | 0.45 | -0.396 | -0.586 | 0.1 | 0.9 |
|  | *A.lyrata (Plech)* | 12 | 0.002 | 0 | 1 | 0.003 | 0 | 0.643 | -1.141 | -0.476 | 0.276* | 0.215 |
|  | *A.lyrata (Pool)* | 72 | 3.00E-04 | 0 | 3.925 | 0.002 | 0 | 0.637 | -1.064 | NA | NA | NA |
| *At2g46550* | *A.halleri* | 60 | 0.014 | 0.01 | 0.435 | 0.013 | 0.01 | 0.403 | 0.178 | -0.154 | 0.105 | 0.627 |
|  | *A.lyrata (Plech)* | 20 | 0.03 | 0 | 0.1 | 0.02 | 0 | 0.086 | 1.650* | 0.866 | 0.211* | 0.88 |
|  | *A.lyrata (Pool)* | 82 | 0.02 | 0 | 0.203 | 0.016 | 0 | 0.11 | 0.560 | NA | NA | NA |
| *At3g20820* | *A.halleri* | 52 | 0.022 | 0 | 0 | 0.018 | 0 | 0.034 | 0.702 | -4.506 | 0.137 | 1.129 |
|  | *A.lyrata (Plech)* | 18 | 0.03 | 0 | 0.1 | 0.021 | 0 | 0.113 | 1.507* | -2.995 | 0.206* | 1.35 |
|  | *A.lyrata (Pool)* | 48 | 0.022 | 0 | 0.136 | 0.018 | 0 | 0.17 | 0.619 | NA | NA | NA |
| *At3g23590* | *A.halleri* | 54 | 0.016 | 0 | 0.19 | 0.013 | 0 | 0.256 | 0.535 | 0.216 | 0.133 | 1.144 |
|  | *A.lyrata (Plech)* | 24 | 0.004 | 0 | 0.75 | 0.004 | 0 | 0.682 | 0.062 | 0.102 | 0.138 | 0.324 |
|  | *A.lyrata (Pool)* | 90 | 0.007 | 0.01 | 0.858 | 0.006 | 0 | 0.508 | 0.363 | NA | NA | NA |
| *At3g48690* | *A.halleri* | 56 | 0.053 | 0.01 | 0.094 | 0.043 | 0.01 | 0.123 | 0.750 | -1.846 | 0.124 | 3.974* |
|  | *A.lyrata (Plech)* | 24 | 0.055 | 0 | 0 | 0.035 | 0 | 0.022 | 2.012* | 1.384* | 0.208* | 2.624 |
|  | *A.lyrata (Pool)* | 80 | 0.045 | 0 | 0.022 | 0.033 | 0 | 0.052 | 1.071 | NA | NA | NA |
| *At3g50740* | *A.halleri* | 54 | 0.005 | 0 | 0.634 | 0.01 | 0 | 0.32 | -1.275* | -3.959* | 0.052* | 0.32 |
|  | *A.lyrata (Plech)* | 22 | 0.011 | 0 | 0.273 | 0.01 | 0 | 0.24 | 0.261 | -1.56 | 0.148 | 0.26 |
|  | *A.lyrata (Pool)* | 84 | 0.049 | 0 | 0.061 | 0.024 | 0 | 0.122 | 2.849 | NA | NA | NA |
| *At3g55060* | *A.halleri* | 50 | 0.009 | 0 | 0.316 | 0.016 | 0.01 | 0.297 | -1.103 | -2.718 | 0.064 | 0.978 |
|  | *A.lyrata (Plech)* | 22 | 0.008 | 0 | 0 | 0.011 | 0 | 0 | -0.736 | -1.542 | 0.106 | 0.915 |
|  | *A.lyrata (Pool)* | 90 | 0.011 | 0 | 0.175 | 0.021 | 0 | 0.078 | -1.151 | NA | NA | NA |
| *At3g62890* | *A.halleri* | 52 | 0.034 | 0 | 0.088 | 0.017 | 0 | 0.111 | 2.737* | 3.029* | 0.191* | 0.581 |
|  | *A.lyrata (Plech)* | 20 | 0.01 | 0.01 | 0.5 | 0.011 | 0 | 0.297 | -0.317 | 1.806 | 0.126 | 0.53 |
|  | *A.lyrata (Pool)* | 74 | 0.051 | 0.01 | 0.216 | 0.03 | 0.01 | 0.196 | 2.077 | NA | NA | NA |
| **Average** | ***A.halleri*** |  | **0.0206** | **0** | **0.563** | **0.017** | **0** | **0.285** | **0.240** | **-0.762** | **0.123** | **NA** |
|  | ***A.lyrata (Plech)*** |  | **0.0228** | **0** | **0.267** | **0.019** | **0** | **0.219** | **0.339** | **0.833** | **0.176** | **NA** |
|  | ***A.lyrata (Pool)*** |  | **0.024** | **0** | **0.532** | **0.019** | **0** | **0.255** | **0.513** | **NA** | **NA** | **NA** |

**P*<0.05*; **P*<0.01; ****P*<0.001
