# Supplementary material for: Does Speciation between Arabidopsis halleri and Arabidopsis lyrata Coincide with Major Changes in a Molecular Target of Adaptation?
Source: PLoS One. 2011 Nov 1;6(11):e26872. doi: 10.1371/journal.pone.0026872 (PMC3206069; doi:10.1371/journal.pone.0026872)
Supplement: Table S4 — Estimates of population nucleotide variation. (DOCX) [file pone.0026872.s009.docx]

| Populations | θ_syn_ | |  | π_syn_ | |
| --- | --- | --- | --- | --- | --- |
|  | Average | Variance |  | Average | Variance |
| Germany | 0.0137 | 0.0003 |  | 0.0160 | 0.0005 |
| CZ | 0.0108 | 0.0002 |  | 0.0114 | 0.0004 |
| France | 0.0123 | 0.0003 |  | 0.0146 | 0.0004 |
| Italy | 0.0130 | 0.0002 |  | 0.0149 | 0.0003 |
| Poland | 0.0142 | 0.0003 |  | 0.0156 | 0.0004 |
| Slovenia | 0.0161 | 0.0003 |  | 0.0176 | 0.0004 |
