# Supplementary material for: Does Speciation between Arabidopsis halleri and Arabidopsis lyrata Coincide with Major Changes in a Molecular Target of Adaptation?
Source: PLoS One. 2011 Nov 1;6(11):e26872. doi: 10.1371/journal.pone.0026872 (PMC3206069; doi:10.1371/journal.pone.0026872)
Supplement: Table S5 — Posterior probabilities of SIC, CMC, AMC and SCC speciation models in four different analyses according to two sample schemes and the two sets of loci, either compared to SIE, CME, AME and SCE models or to SIB, CMB, AMB and SCB models. (DOCX) [file pone.0026872.s010.docx]

| **Analysis** | **Scenario** | **Posterior probabilities of constant population size models against :** | | **Posterior probabilities of the best models selected under each scenario** |
| --- | --- | --- | --- | --- |
|  |  | **exponential population growth model** | **recent botleneck specific to *A. halleri*** |  |
| Plech19 | SI | 0.585 | 0.608 | 0.38 |
|  | CM | 0.6 | 0.635 | 0.055 |
|  | AM | 0.749 | 0.552 | **0.439** |
|  | SC | 0.632 | 0.577 | 0.127 |
| Plech28 | SI | 0.525 | 0.581 | **0.719** |
|  | CM | 0.561 | 0.676 | 0.001 |
|  | AM | 0.755 | 0.743 | 0.279 |
|  | SC | 0.503 | 0.51 | 0.001 |
| Pool19 | SI | 0.827 | 0.644 | **0.824** |
|  | CM | 0.684 | 0.797 | 0.011 |
|  | AM | 0.8 | 0.685 | 0.142 |
|  | SC | 0.63 | 0.734 | 0.023 |
| Pool28 | SI | 0.623 | 0.732 | **0.771** |
|  | CM | 0.699 | 0.831 | 0.001 |
|  | AM | 0.714 | 0.833 | 0.227 |
|  | SC | 0.652 | 0.736 | 0.001 |
