# Supplementary material for: Does Speciation between Arabidopsis halleri and Arabidopsis lyrata Coincide with Major Changes in a Molecular Target of Adaptation?
Source: PLoS One. 2011 Nov 1;6(11):e26872. doi: 10.1371/journal.pone.0026872 (PMC3206069; doi:10.1371/journal.pone.0026872)
Supplement: Table S6 — Summary statistics used in the different procedures of the ABC analysis. (DOCX) [file pone.0026872.s011.docx]

|  | **Biallelic Positions** | | ***S*f*_hal_*** | | ***S*f*_lyr_*** | | ***S*x*_hal_*** | | ***S*x*_lyr_*** | | ***S*x*_hal_*f*_lyr_*** | | ***S*x*_lyr_*f*_hal_*** | | ***S*s** | | **π_hal_** | | **π_lyr_** | | **θ_hal_** | | **θ_lyr_** | | **Tajima's *D* in *A. halleri*** | | **Tajima's *D* *A. lyrata*** | | **Gross Divergence** | | **Net Divergence** | | **F_ST_** | |
| --- | --- | --- | --- | --- | --- | --- | --- | --- | --- | --- | --- | --- | --- | --- | --- | --- | --- | --- | --- | --- | --- | --- | --- | --- | --- | --- | --- | --- | --- | --- | --- | --- | --- | --- |
|  | **avg** | **sd** | **avg** | **sd** | **avg** | **sd** | **avg** | **sd** | **avg** | **sd** | **avg** | **sd** | **avg** | **sd** | **avg** | **sd** | **avg** | **sd** | **avg** | **sd** | **avg** | **sd** | **avg** | **sd** | **avg** | **sd** | **avg** | **sd** | **avg** | **sd** | **avg** | **sd** | **avg** | **sd** |
| **Model choice** | Used | Used | Used | Used | Used | Used | Used | Used | Used | Used | Used | Used | Used | Used | Used | Used | Not Used | Not Used | Not Used | Not Used | Not Used | Not Used | Not Used | Not Used | Used | Used | Used | Used | Not Used | Not Used | Not Used | Not Used | Used | Used |
| **Estimation of parameters** | Used | Used | Used | Used | Used | Used | Used | Used | Used | Used | Used | Used | Used | Used | Used | Used | Not Used | Not Used | Not Used | Not Used | Not Used | Not Used | Not Used | Not Used | Used | Used | Used | Used | Not Used | Not Used | Not Used | Not Used | Used | Used |
| **Goodness-of-fit** | Used | Used | Used | Used | Used | Used | Used | Used | Used | Used | Used | Used | Used | Used | Used | Used | Used | Used | Used | Used | Used | Used | Used | Used | Used | Used | Used | Used | Used | Used | Used | Used | Used | Used |
