# Supplementary material for: Does Speciation between Arabidopsis halleri and Arabidopsis lyrata Coincide with Major Changes in a Molecular Target of Adaptation?
Source: PLoS One. 2011 Nov 1;6(11):e26872. doi: 10.1371/journal.pone.0026872 (PMC3206069; doi:10.1371/journal.pone.0026872)
Supplement: Table S7 — Results (P-values) of the goodness-of-fit tests for the SIC and AMC models for each of the four datasets. (DOCX) [file pone.0026872.s012.docx]

| **Analysis** | **Models** | **Bialsites** | | ***S*f*_hal_*** | | ***S*f*_lyr_*** | | ***S*x*_hal_*** | | ***S*x*_lyr_*** | | ***S*x*_hal_*f*_lyr_*** | | ***S*x*_hal_*f*_lyr_*** | | ***S*s** | | **π*_hal_*** | | **π*_lyr_*** | | **θ*_hal_*** | | **θ*_lyr_*** | | **Tajima’s *D_hal_*** | | **Tajima’s *D_lyr_*** | | **Gross divergence** | | **Net divergence** | | **F_ST_** | |
| --- | --- | --- | --- | --- | --- | --- | --- | --- | --- | --- | --- | --- | --- | --- | --- | --- | --- | --- | --- | --- | --- | --- | --- | --- | --- | --- | --- | --- | --- | --- | --- | --- | --- | --- | --- |
|  |  | **avg** | **std** | **avg** | **std** | **avg** | **std** | **avg** | **std** | **avg** | **std** | **avg** | **std** | **avg** | **std** | **avg** | **std** | **avg** | **std** | **avg** | **std** | **avg** | **std** | **vg** | **std** | **avg** | **std** | **avg** | **std** | **avg** | **std** | **avg** | **std** | **avg** | **std** |
| **plech19** | **SIC** | 0.38 | 0.17 | 0.36 | 0.16 | 0.5 | 0.24 | 0.35 | 0.203 | 0.44 | 0.48 | 0.37 | 0.351 | 0.18 | 0.21 | 0.45 | 0.27 | 0.261 | 0.211 | 0.45 | 0.35 | 0.43 | 0.2 | 0.43 | 0.12 | 0.079 | 0.15 | 0.16 | 0.35 | 0.4 | 0.05 | 0.49 | 0.067 | 0.25 | 0.01 |
|  | **AMC** | 0.42 | 0.16 | 0.46 | 0.34 | 0.32 | 0.34 | 0.23 | 0.17 | 0.28 | 0.4 | 0.37 | 0.232 | 0.16 | 0.15 | 0.33 | 0.21 | 0.265 | 0.202 | 0.47 | 0.46 | 0.41 | 0.1 | 0.33 | 0.31 | 0.104 | 0.19 | 0.13 | 0.36 | 0.43 | 0.1 | 0.42 | 0.119 | 0.19 | 0.05 |
| **plech28** | **SIC** | 0.45 | 0.07 | 0.4 | 0.24 | 0.43 | 0.27 | 0.32 | 0.31 | 0.48 | 0.37 | 0.46 | 0.237 | 0.13 | 0.1 | 0.4 | 0.14 | 0.222 | 0.234 | 0.33 | 0.48 | 0.34 | 0.26 | 0.46 | 0.15 | 0.098 | 0.26 | 0.43 | 0.19 | 0.41 | 0.19 | 0.35 | 0.232 | 0.42 | 0.12 |
|  | **AMC** | 0.35 | 0.29 | 0.33 | 0.46 | 0.25 | 0.43 | 0.19 | 0.445 | 0.45 | 0.46 | 0.33 | 0.431 | 0.19 | 0.15 | 0.36 | 0.14 | 0.415 | 0.441 | 0.39 | 0.42 | 0.39 | 0.39 | 0.4 | 0.3 | 0.262 | 0.44 | 0.43 | 0.14 | 0.23 | 0.34 | 0.25 | 0.436 | 0.2 | 0.35 |
| **pool19** | **SIC** | 0.21 | 0.18 | 0.49 | 0.21 | 0.47 | 0.31 | 0.19 | *0.02* | 0.49 | 0.4 | 0.28 | 0.066 | 0.14 | 0.31 | 0.37 | 0.08 | *0.03* | *0.02* | 0.26 | 0.25 | 0.18 | *0.02* | 0.44 | 0.1 | *0.02* | 0.06 | 0.12 | 0.17 | 0.19 | *0.01* | 0.33 | *0.01* | 0.05 | 0.25 |
|  | **AMC** | 0.42 | 0.31 | 0.28 | 0.48 | 0.28 | 0.46 | 0.47 | 0.104 | 0.39 | 0.35 | 0.43 | 0.279 | 0.22 | 0.26 | 0.45 | 0.13 | 0.268 | 0.129 | 0.38 | 0.44 | 0.43 | 0.08 | 0.46 | 0.29 | 0.094 | 0.12 | 0.19 | *0.04* | 0.34 | 0.09 | 0.3 | 0.099 | 0.07 | 0.33 |
| **pool28** | **SIC** | 0.31 | 0.26 | 0.47 | 0.31 | 0.47 | 0.34 | 0.24 | 0.068 | 0.31 | 0.3 | 0.41 | *0.04* | 0.09 | 0.11 | 0.34 | 0.11 | 0.202 | 0.433 | 0.48 | 0.29 | 0.45 | 0.37 | 0.46 | 0.47 | 0.086 | 0.18 | 0.27 | 0.33 | 0.18 | 0.06 | 0.21 | 0.066 | 0.3 | 0.36 |
|  | **AMC** | 0.32 | 0.38 | 0.36 | 0.39 | 0.26 | 0.4 | 0.31 | 0.26 | 0.33 | 0.37 | 0.33 | 0.17 | 0.2 | 0.31 | 0.48 | 0.21 | 0.455 | 0.464 | 0.32 | 0.29 | 0.41 | 0.28 | 0.44 | 0.5 | 0.19 | 0.22 | 0.44 | 0.14 | 0.37 | 0.12 | 0.44 | 0.203 | 0.16 | 0.35 |
