# Supplementary material for: Does Speciation between Arabidopsis halleri and Arabidopsis lyrata Coincide with Major Changes in a Molecular Target of Adaptation?
Source: PLoS One. 2011 Nov 1;6(11):e26872. doi: 10.1371/journal.pone.0026872 (PMC3206069; doi:10.1371/journal.pone.0026872)
Supplement: Table S8 — Levels per locus of synonymous (Ksyn) and non-synonymous (Kasyn) divergence among Arabidopsis species, estimates of FST per locus and mutation rates per bp per generation assuming a divergence time of 5 MY with A. thaliana. (DOCX) [file pone.0026872.s013.docx]

| Locus | Pair | K_syn_ | K_asyn_ | K_asyn_/K_syn_ | F_ST_ | µ_syn_ |
| --- | --- | --- | --- | --- | --- | --- |
| *At1g01040* | *Arabidopsis halleri* versus *Arabidopsis lyrata* | 0.049 | 0.002 | 0.041 | 0.16 |  |
|  | *A. halleri* versus *A. thaliana* | 0.133 | 0.006 | 0.047 |  | 2.25E-08 |
|  | *A. lyrata* versus *A. thaliana* | 0.145 | 0.009 | 0.059 |  |  |
| *At1g03560* | *A. halleri* versus *A. lyrata* | 0.103 | 0.011 | 0.107 | 0.44 |  |
|  | *A.* *halleri* versus *A. thaliana* | 0.192 | 0.025 | 0.129 |  | 2.85E-08 |
|  | *A. lyrata* versus *A. thaliana* | 0.152 | 0.022 | 0.146 |  |  |
| *At1g04650* | *A. halleri* versus *A. lyrata* | 0.029 | 0.012 | 0.413 | 0.74 |  |
|  | *A.* *halleri* versus *A. thaliana* | 0.111 | 0.017 | 0.151 |  | 1.83E-08 |
|  | *A. lyrata* versus *A. thaliana* | 0.079 | 0.019 | 0.241 |  |  |
| *At1g06520* | *A. halleri* versus *A. lyrata* | 0.065 | 0.004 | 0.061 | 0.69 |  |
|  | *A.* *halleri* versus *A. thaliana* | 0.151 | 0.032 | 0.211 |  | 3.12E-08 |
|  | *A. lyrata* versus *A. thaliana* | 0.175 | 0.036 | 0.205 |  |  |
| *At1g06530* | *A. halleri* versus *A. lyrata* | 0.025 | 0.008 | 0.313 | 0.59 |  |
|  | *A.* *halleri* versus *A. thaliana* | 0.109 | 0.035 | 0.324 |  | 2.25E-08 |
|  | *A. lyrata* versus *A. thaliana* | 0.129 | 0.041 | 0.318 |  |  |
| *At1g10900* | *A. halleri* versus *A. lyrata* | 0.029 | 0.005 | 0.159 | 0.19 |  |
|  | *A.* *halleri* versus *A. thaliana* | 0.099 | 0.015 | 0.15 |  | 1.77E-08 |
|  | *A. lyrata* versus *A. thaliana* | 0.109 | 0.012 | 0.113 |  |  |
| *At1g10980* | *A. halleri* versus *A. lyrata* | 0.049 | 0.016 | 0.326 | 0.33 |  |
|  | *A.* *halleri* versus *A. thaliana* | 0.077 | 0.039 | 0.502 |  | 1.58E-08 |
|  | *A. lyrata* versus *A. thaliana* | 0.111 | 0.032 | 0.286 |  |  |
| *At1g11050* | *A. halleri* versus *A. lyrata* | 0.063 | 0.017 | 0.265 | 0.55 |  |
|  | *A.* *halleri* versus *A. thaliana* | 0.22 | 0.016 | 0.073 |  | 3.74E-08 |
|  | *A. lyrata* versus *A. thaliana* | 0.196 | 0.018 | 0.093 |  |  |
| *At1g15240* | *A. halleri* versus *A. lyrata* | 0.131 | 0.024 | 0.183 | 0.87 |  |
|  | *A.* *halleri* versus *A. thaliana* | 0.119 | 0.033 | 0.279 |  | 2.82E-08 |
|  | *A. lyrata* versus *A. thaliana* | 0.177 | 0.033 | 0.187 |  |  |
| *At1g59720* | *A. halleri* versus *A. lyrata* | 0.097 | 0.016 | 0.162 | 0.04 |  |
|  | *A.* *halleri* versus *A. thaliana* | 0.157 | 0.055 | 0.351 |  | 1.34E-08 |
|  | *A. lyrata* versus *A. thaliana* | 0.151 | 0.058 | 0.387 |  |  |
| *At1g62310* | *A. halleri* versus *A. lyrata* | 0.051 | 0.006 | 0.124 | 0.33 |  |
|  | *A.* *halleri* versus *A. thaliana* | 0.144 | 0.022 | 0.154 |  | 2.22E-08 |
|  | *A. lyrata* versus *A. thaliana* | 0.127 | 0.024 | 0.187 |  |  |
| *At1g62390* | *A. halleri* versus *A. lyrata* | 0.065 | 0.006 | 0.087 | 0.28 |  |
|  | *A.* *halleri* versus *A. thaliana* | 0.156 | 0.014 | 0.09 |  | 2.11E-08 |
|  | *A. lyrata* versus *A. thaliana* | 0.144 | 0.016 | 0.112 |  |  |
| *At1g62520* | *A. halleri* versus *A. lyrata* | 0.069 | 0.004 | 0.062 | 0.32 |  |
|  | *A.* *halleri* versus *A. thaliana* | 0.259 | 0.022 | 0.085 |  | 4.67E-08 |
|  | *A. lyrata* versus *A. thaliana* | 0.285 | 0.022 | 0.078 |  |  |
| *At1g64170* | *A. halleri* versus *A. lyrata* | 0.064 | 0.006 | 0.093 | 0.51 |  |
|  | *A.* *halleri* versus *A. thaliana* | 0.116 | 0.016 | 0.136 |  | 2.45E-08 |
|  | *A. lyrata* versus *A. thaliana* | 0.17 | 0.019 | 0.11 |  |  |
| *At1g72390* | *A. halleri* versus *A. lyrata* | 0 | 0.006 | NA | NA |  |
|  | *A.* *halleri* versus *A. thaliana* | 0.125 | 0.028 | 0.224 |  | 2.5E-08 |
|  | *A. lyrata* versus *A. thaliana* | 0.125 | 0.029 | 0.23 |  |  |
| *At1g74600* | *A. halleri* versus *A. lyrata* | 0.071 | 0.01 | 0.141 | NA |  |
|  | *A.* *halleri* versus *A. thaliana* | 0.175 | 0.027 | 0.157 |  | 2.71E-08 |
|  | *A. lyrata* versus *A. thaliana* | 0.185 | 0.028 | 0.153 |  |  |
| *At2g16870* | *A. halleri* versus *A. lyrata* | 0.073 | 0.017 | 0.236 | 0.34 |  |
|  | *A.* *halleri* versus *A. thaliana* | 0.148 | 0.05 | 0.335 |  | 2.34E-08 |
|  | *A. lyrata* versus *A. thaliana* | 0.141 | 0.045 | 0.318 |  |  |
| *At2g23170* | *A. halleri* versus *A. lyrata* | 0.091 | 0.006 | 0.069 | 0.42 |  |
|  | *A.* *halleri* versus *A. thaliana* | 0.124 | 0.015 | 0.124 |  | 2.71E-08 |
|  | *A. lyrata* versus *A. thaliana* | 0.199 | 0.014 | 0.072 |  |  |
| *At2g26140* | *A. halleri* versus *A. lyrata* | 0.015 | 0 | 0 | 0.66 |  |
|  | *A.* *halleri* versus *A. thaliana* | 0.136 | 0 | 0 |  | 2.49E-08 |
|  | *A. lyrata* versus *A. thaliana* | 0.119 | 0 | 0 |  |  |
| *At2g26730* | *A. halleri* versus *A. lyrata* | 0.037 | 0 | 0 | 0.43 |  |
|  | *A.* *halleri* versus *A. thaliana* | 0.246 | 0.004 | 0.016 |  | 4.52E-08 |
|  | *A. lyrata* versus *A. thaliana* | 0.243 | 0.004 | 0.016 |  |  |
| *At2g43680* | *A. halleri* versus *A. lyrata* | 0.048 | 0.006 | 0.122 | 0.24 |  |
|  | *A.* *halleri* versus *A. thaliana* | 0.154 | 0.01 | 0.063 |  | 2.48E-08 |
|  | *A. lyrata* versus *A. thaliana* | 0.123 | 0.012 | 0.095 |  |  |
| *At2g44900* | *A. halleri* versus *A. lyrata* | 0.034 | 0.007 | 0.214 | 0.68 |  |
|  | *A.* *halleri* versus *A. thaliana* | 0.104 | 0.021 | 0.203 |  | 2.05E-08 |
|  | *A. lyrata* versus *A. thaliana* | 0.111 | 0.017 | 0.152 |  |  |
| *At2g46550* | *A. halleri* versus *A. lyrata* | 0.083 | 0.013 | 0.157 | 0.47 |  |
|  | *A.* *halleri* versus *A. thaliana* | 0.161 | 0.038 | 0.238 |  | 3.16E-08 |
|  | *A. lyrata* versus *A. thaliana* | 0.198 | 0.032 | 0.16 |  |  |
| *At3g20820* | *A. halleri* versus *A. lyrata* | 0.08 | 0.003 | 0.032 | 0.42 |  |
|  | *A.* *halleri* versus *A. thaliana* | 0.137 | 0.004 | 0.027 |  | 2.31E-08 |
|  | *A. lyrata* versus *A. thaliana* | 0.146 | 0.002 | 0.015 |  |  |
| *At3g23590* | *A. halleri* versus *A. lyrata* | 0.014 | 0.009 | 0.616 | 0.3 |  |
|  | *A.* *halleri* versus *A. thaliana* | 0.12 | 0.023 | 0.195 |  | 2.2E-08 |
|  | *A. lyrata* versus *A. thaliana* | 0.12 | 0.032 | 0.266 |  |  |
| *At3g48690* | *A. halleri* versus *A. lyrata* | 0.115 | 0.015 | 0.129 | 0.32 |  |
|  | *A.* *halleri* versus *A. thaliana* | 0.153 | 0.015 | 0.101 |  | 2.18E-08 |
|  | *A. lyrata* versus *A. thaliana* | 0.174 | 0.024 | 0.138 |  |  |
| *At3g50740* | *A. halleri* versus *A. lyrata* | 0.018 | 0.005 | 0.251 | 0.27 |  |
|  | *A.* *halleri* versus *A. thaliana* | 0.228 | 0.017 | 0.074 |  | 4.6E-08 |
|  | *A. lyrata* versus *A. thaliana* | 0.248 | 0.017 | 0.069 |  |  |
| *At3g55060* | *A. halleri* versus *A. lyrata* | 0.038 | 0.006 | 0.148 | 0.58 |  |
|  | *A.* *halleri* versus *A. thaliana* | 0.139 | 0.008 | 0.06 |  | 2.27E-08 |
|  | *A. lyrata* versus *A. thaliana* | 0.106 | 0.009 | 0.086 |  |  |
| *At3g62890* | *A. halleri* versus *A. lyrata* | 0.128 | 0.029 | 0.228 | 0.68 |  |
|  | *A.* *halleri* versus *A. thaliana* | 0.27 | 0.037 | 0.137 |  | 4.32E-08 |
|  | *A. lyrata* versus *A. thaliana* | 0.207 | 0.032 | 0.153 |  |  |
| ***Average*** | ***A. halleri* versus *A. lyrata*** | **0.06** | **0.009** | **0.154** | **0.438** |  |
|  | ***A.* *halleri* versus *A. thaliana*** | **0.154** | **0.022** | **0.145** |  | 2.68E-08 |
|  | ***A. lyrata* versus *A. thaliana*** | **0.158** | **0.023** | **0.143** |  |  |
